# Association of Obesity With Septic Complications After Major Abdominal Surgery: A Secondary Analysis of the RELIEF Randomized Clinical Trial

**DOI:** 10.1001/jamanetworkopen.2019.16345

**Published:** 2019-11-27

**Authors:** Usha Gurunathan, Ivan L. Rapchuk, Marilla Dickfos, Peter Larsen, Andrew Forbes, Catherine Martin, Kate Leslie, Paul S. Myles

**Affiliations:** 1 Department of Anaesthesia and Perfusion Services, The Prince Charles Hospital, Brisbane, Queensland, Australia; 2 School of Medicine, University of Queensland, Brisbane, Queensland, Australia; 3 Department of General Surgery, Rockhampton Hospital, Rockhampton, Queensland, Australia; 4 School of Public Health and Preventive Medicine, Monash University, Melbourne, Victoria, Australia; 5 Department of Anaesthesia and Pain Management, Royal Melbourne Hospital, Melbourne, Victoria, Australia; 6 Centre for Integrated Critical Care Medicine, Melbourne Medical School, University of Melbourne, Melbourne, Victoria, Australia; 7 Department of Pharmacology and Therapeutics, University of Melbourne, Melbourne, Victoria, Australia; 8 Department of Anaesthesiology and Perioperative Medicine, Alfred Hospital and Monash University, Melbourne, Victoria, Australia

## Abstract

**Importance:**

Body mass index (BMI) has been the most common obesity measure to assess perioperative risk; however, cardiometabolic risk is associated with the burden of visceral fat. Definitive evidence on the association of visceral fat measures, such as waist circumference and waist-to-hip ratio (WHR), with postoperative complications is lacking.

**Objective:**

To compare the value of waist circumference with the value WHR and BMI in predicting adverse outcomes, including major septic complications and persistent disability, following major abdominal surgery.

**Design, Setting, and Participants:**

This planned secondary analysis of the Restrictive vs Liberal Fluid Therapy for Major Abdominal Surgery (RELIEF) randomized clinical trial took place at 47 centers in 7 countries between October 2013 and September 2016, with 90-day follow-up. A total of 2954 adult RELIEF participants were coenrolled in this secondary analysis. Data analysis took place from December 2018 to September 2019.

**Exposures:**

Waist circumference, WHR, and BMI measurements.

**Main Outcomes and Measures:**

The primary outcomes were 30-day major septic complications and 90-day persistent disability or death.

**Results:**

Of 2954 eligible participants, 2755 were included (mean [SD] age, 65.9 [12.9] years; 1426 [51.8%] men) in the final analysis. A total of 564 participants (20.6%) experienced at least 1 major septic complication within 30 days after surgery (sepsis, 265 [9.7%]; surgical site infection, 409 [14.9%]; anastomotic leak, 78 [2.8%]; pneumonia, 104 [3.8%]). Waist circumference had a statistically significantly larger odds ratio (OR) and discrimination indices as well as a smaller prediction error than WHR or BMI for 30-day major septic complications or death (waist circumference: OR, 1.44; 95% CI, 1.28-1.62; *P* < .001; area under the receiver operating characteristic curve, 0.641; net reclassification index, 0.266; integrated discrimination improvement [score × 10^4^], 152.98; Brier score, 0.162; WHR: OR, 1.15; 95% CI, 1.03-1.28; *P* = .01; area under the receiver operating characteristic curve, 0.621; net classification index, 0.199; integrated discrimination improvement [score × 10^4^], 28.47; Brier score, 0.164; BMI: OR, 1.33; 95% CI, 1.17-1.50; *P* < .001; area under the receiver operating characteristic curve, 0.629; net reclassification index, 0.205; integrated discrimination improvement [score × 10^4^], 85.61; Brier score, 0.163) but not for any other outcomes.

**Conclusions and Relevance:**

In this secondary analysis of the RELIEF randomized clinical trial, waist circumference was observed to be superior to other adiposity indices in predicting 30-day major septic complications alone or in conjunction with death following elective major abdominal surgery. Findings suggest that waist circumference is a useful adiposity measure that should be incorporated in preoperative risk assessment for such complications.

**Trial Registration:**

ClinicalTrials.gov identifier: NCT01424150

## Key Points

**Question:**

Is waist circumference more predictive of adverse outcomes following major abdominal surgery than body mass index and waist-to-hip ratio?

**Findings:**

In this secondary analysis of the Restrictive vs Liberal Fluid Therapy for Major Abdominal Surgery (RELIEF) randomized clinical trial involving 2954 adult patients, it was observed that waist circumference was the superior predictor compared with body mass index and waist-to-hip ratio for 30-day major septic complications alone or in conjunction with death following elective major abdominal surgery.

**Meaning:**

The findings of this study suggest that waist circumference is a useful measure in the preoperative risk prediction for septic complications following elective major abdominal surgery.

## Teaser

This secondary analysis of the Restrictive vs Liberal Fluid Therapy for Major Abdominal Surgery (RELIEF) randomized clinical trial compares the value of waist circumference with the value of waist-to-hip ratio and body mass index in predicting adverse outcomes, including major septic complications and persistent disability, following major abdominal surgery.

## Introduction

Obesity is considered a risk factor for infective complications in most surgical procedures.^1,2^ Postoperative sepsis is associated with substantial morbidity and mortality. In fact, it has been shown to be an independent predictor of mortality up to a year after elective surgery.^3^ Thelwall et al^2^ reported 1.1-fold to 4.4-fold higher odds of developing surgical site infection (SSI) in patients with overweight and obesity compared with patients with normal weight. Among patients who underwent colorectal surgery, the odds of SSI have been found to be 20% higher in individuals with overweight compared with those with normal weight and 50% higher in individuals with obesity compared with those without obesity.^4^

Obesity is generally defined as excess body fat, with body mass index (BMI, calculated as weight in kilograms divided by height in meters squared) being the most widely used clinical measure.^5^ However, despite being useful for population-level studies, BMI has its drawbacks.^6^ One drawback is that BMI is not consistently associated with postoperative complications.^7,8,9^ Another is that obesity is a heterogeneous condition with variable cardiovascular risk in individuals with similar BMI.^10^ Furthermore, BMI may not correlate well with body fat composition.^11^ When compared with other adiposity measures, the association of BMI with cardiovascular disease is more susceptible to confounding because of comorbidities and smoking.^12^

Visceral adiposity is considered a good marker of altered cardiometabolic risk profile, and measures of central obesity, such as waist circumference and waist-to-hip ratio (WHR), have been recognized as useful perioperative risk assessment tools.^13,14^ To our knowledge, very few studies have assessed the perioperative SSI risk in abdominal surgery associated with increasing waist circumference. One of them, a retrospective review,^15^ showed that waist circumference was better than BMI at predicting short-term complications in patients undergoing elective rectal resections. Studies on visceral adiposity have been single-centered, conducted on small samples of patients, and limited to specific surgery types. In addition, many of the studies were undertaken in the Asian population, limiting their generalizability. We proposed the current study to estimate the value of waist circumference in predicting postoperative adverse outcomes and comparing it with that of BMI and WHR in patients undergoing major abdominal surgery. We use the broad term *value* to indicate performance over a range of utility indices, as follows: association with outcomes, ability to discriminate between outcome events and nonevents, incremental improvement in prediction, and average squared prediction error.

We hypothesized that surgical patients with increased waist circumference would have increased 30-day postoperative complications and increased 90-day disability or death and that waist circumference would have greater value as a predictor of adverse outcomes than BMI. This project was a planned substudy of the international multicenter Restrictive vs Liberal Fluid Therapy for Major Abdominal Surgery (RELIEF) trial.^16^

## Methods

The RELIEF trial was an international 1:1 randomized multicenter trial comparing a restrictive intravenous fluid regimen with a liberal intravenous fluid regimen in patients who underwent major abdominal surgery.^16,17^ The trial protocol is available in Supplement 1. We coenrolled RELIEF participants in our substudy and performed secondary analyses of the relevant data. The participants included adult patients undergoing elective major abdominal surgery of at least 2 hours’ duration, with a hospital stay of at least 3 days, and an increased risk of postoperative complications. Detailed inclusion and exclusion criteria are provided in eTable 1 in Supplement 2.^16^ The substudy was approved by ethics committees at participating sites, and all participants provided written informed consent. This study followed the Strengthening the Reporting of Observational Studies in Epidemiology (STROBE) reporting guideline.

For the RELIEF trial, the liberal intravenous fluid regimen included a bolus of balanced salt crystalloid solution of 10 mL/kg at induction followed by 8 mL/kg/h until the end of surgery. The restrictive intravenous fluid regimen included a bolus dose of balanced salt crystalloid solution of 5 mL/kg at induction followed by 5 mL/kg/h until the end of surgery. The postoperative fluid rate was 1.5 mL/kg/h in the liberal arm and 0.8 mL/kg/h in the restrictive arm for at least the first 24 hours, which could be altered according to the volume status and hemodynamic condition of the patients.^16^ Waist circumference and WHR were measured in addition to the other data collected for the main study. All participating centers were instructed to measure waist circumference in centimeters using a measuring tape placed horizontally midway between the lowest rib and top of iliac crest, while hip circumference was measured in centimeters around the widest portion of the gluteal region with the tape parallel to the floor. Waist-to-hip ratio was calculated as waist circumference divided by hip circumference.

### Outcomes

The primary outcomes of this substudy were as follows: (1) 30-day major septic complications (ie, sepsis, SSI, anastomotic leak, and pneumonia) and (2) persistent disability or death by 90 days. Persistent disability was defined as a World Health Organization Disability Assessment Schedule 2.0^18^ questionnaire score of at least 24 points (on the 48-point scale) at both 30 days and 90 days after the operation. Secondary outcomes for this substudy were a prospectively defined composite of 30-day mortality or any major septic complication (ie, sepsis, SSI, anastomotic leak, and pneumonia) with pulmonary edema or acute kidney injury to 30 days (acute kidney injury measured by at least a doubling of preoperative serum creatinine or a halving of estimated glomerular filtration rate),^19^ unplanned admission to intensive care unit (ICU) to 30 days, and Quality of Recovery 15 score^20^ at 30 days. To ascertain outcomes, participants were observed during their hospital admission, their medical were records reviewed, source documentation was verified, and participants were contacted by research staff.

### Statistical Analysis

The analyses were performed in a modified intention-to-treat population, which included all participants who had undergone both randomization and induction of general anesthesia. The initial phase of the analysis explored the nature of the relationship of the 3 adiposity measures (ie, waist circumference, BMI, and WHR) with the 7 outcome variables. To allow potential nonlinearities in these relationships, a method called *fractional polynomials* was used.^21,22^ In brief, the method performs a systematic search over a wide range of nonlinear functions of the adiposity measures, including functional shapes with a maximum point (ie, a hump) or a variety of threshold effects, chooses the best fitting model, and compares this model with that of a model with a linear term for the adiposity variable. All models were adjusted for a preselected set of confounding covariates as fixed effects, as follows: age, sex, smoking status, allocation (ie, restrictive or liberal fluids), and study center. The incorporation of the latter in the models ensured that all adiposity-outcomes were assessed within each center. Centers that had 0 events for a particular outcome did not provide information on within-center adiposity associations with that outcome. Therefore, patients from those centers were excluded from the analyses for that outcome.

For the 6 binary outcomes, the best-fitting model was used in a logistic regression to calculate an odds ratio (OR) per SD increase in the adiposity predictor, together with its 95% CI and *P* value. The presentation in terms of SD units enabled direct comparison of the ORs of the 3 adiposity measures for each outcome. We calculated 4 additional indices of the value of each adiposity variable, as follows: (1) area under the receiver operating characteristic curve (AUC), a measure of the ability of the model with a particular adiposity variable to discriminate between outcome events and nonevents; (2) net reclassification index (NRI),^23^ which measures the incremental value of adding the variable to an existing model (ie, that of the 4 adjustment covariates plus study center); (3) integrated discrimination improvement (IDI), which compares the discrimination slope between models with and without the variable, in which the discrimination slope is the average predicted risk in patients with the outcome minus the average predicted risk in patients without the outcome; and (4) the Brier Score, which is the mean-squared prediction error and thus a direct assessment of model fit.^24^ For the continuously valued quality of recovery score, the best-fitting model for each of the 3 adiposity variables was compared using linear regression with standardized regression coefficients and the conventional *R*^2^ statistic.

To address whether differences between the indices of value of each adiposity predictor were greater than expected by chance variation, we used a bootstrap procedure to calculate bootstrap estimates of *P* values for each pairwise comparison of adiposity measures. We performed this by resampling from the source data set to create 10 000 bootstrap samples. For each sample, we calculated each value index for each adiposity variable and each outcome and the difference in each value index between each pair of adiposity variables. This produced 10 000 bootstrap replications of each of these pairwise differences, from which we computed bootstrap percentile CIs for each pairwise difference. Using the analogy between the level of a CI and that of a significance test (eg, 95% CI and *P* < .05), we iteratively calculated the confidence level required so that the bootstrap percentile CI just touched the 0 point, from which the bootstrap *P* value was calculated as 1 minus the CI level divided by 100. In this manner, we obtained *P* values for comparing each value index between each pair of adiposity variables for each outcome.

Graphs of the relationship between each predictor and the outcome for the best-fitting model were produced, adjusting for age, sex, smoking status, and treatment arm by fixing these variables at age (mean value, 66.1 years), sex (men), smoking status (nonsmoker), and treatment arm (liberal arm) and at study center 1. Graphs for other study centers were shifted higher or lower but maintained the same shape (ie, the same slope on the logit scale). All the analyses were performed using Stata version 15 (StataCorp). A 2-tailed *P* < .05 was considered statistically significant.

## Results

A total of 2954 patients met the modified intention-to-treat definition from October 2013 to September 2016. Of these patients, 197 (0.1%) had missing information on at least 1 adiposity measure, and they were excluded. There were 2 study centers that enrolled only 1 patient each, thereby preventing assessment of within-center associations between adiposity and outcomes, and they were therefore excluded. This left 2755 participants with complete data on waist circumference, WHR, and BMI, and all analyses were restricted to this set of participants (Figure). Of these patients, 2742 (99.6%) had data on septic complications and pulmonary edema at 30 days, 2683 (97.5%) had data on death and disability at 90 days, 2663 (96.7%) had data on acute kidney injury, 2751 (99.9%) had data on unplanned intensive care unit admission at 30 days, and 2668 (96.9%) had data on quality of recovery at 30 days. Of these, we included 2636 (96.1%) with data on septic complications, 1695 (61.8%) with data on pulmonary edema, 2661 (99.2%) with data on death or disability to 90 days, 2598 (97.6%) with data on acute kidney injury, 2661 (96.7%) with data on unplanned ICU admission at 30 days, and 2668 (100%) with data on quality of recovery at 30 days in the analysis.

**Figure.**
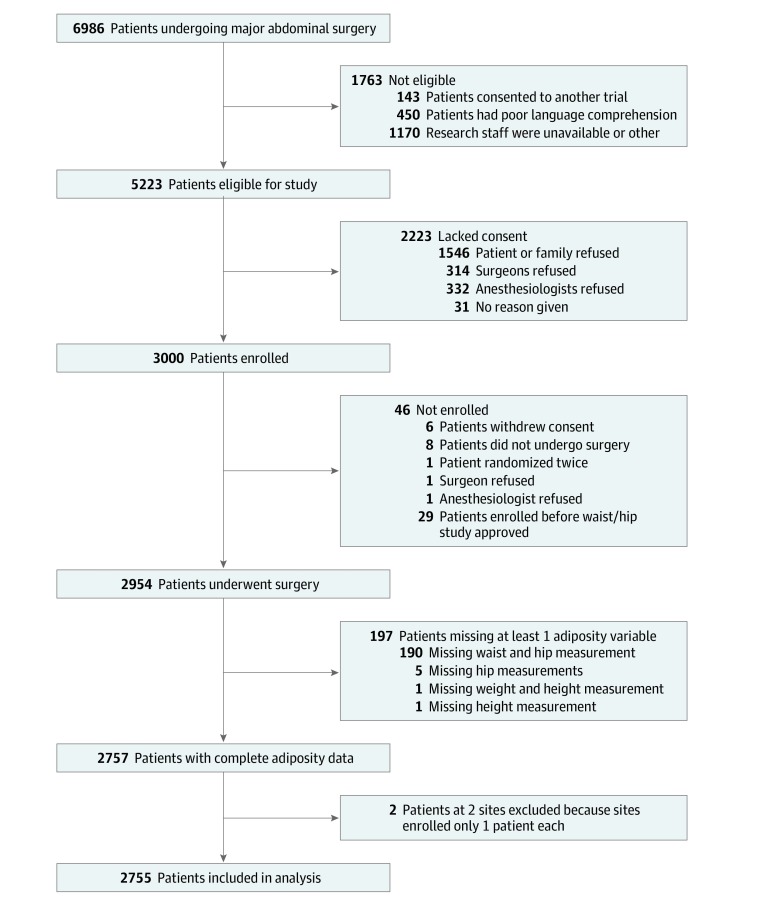
Study Flow Diagram Of 2755 eligible patients, 2742 (99.6%) had data on septic complications and pulmonary edema at 30 days, 2683 (97.5%) had data on death and disability at 90 days, 2663 (96.7%) had data on acute kidney injury, 2751 (99.9%) had data on unplanned intensive care unit admission at 30 days, and 2668 (96.9%) had data on quality of recovery at 30 days. Of these, 2636 (96.1%) with data on septic complications, 1695 (61.8%) with data on pulmonary edema, 2661 (99.2%) with data on death or disability to 90 days, 2598 (97.6%) with data on acute kidney injury, 2661 (96.7%) with data on unplanned intensive care unit admission at 30 days, and 2668 (100%) with data on quality of recovery at 30 days were included in the analysis.

Summary statistics of the adiposity variables are presented in Table 1, and histograms showing the distribution of the adiposity variables are presented in eFigure 1 in Supplement 2. The mean (SD) age of participants was 65.9 (12.9) years; 1426 (51.8%) were men, 365 (13.3%) were current smokers, and 1381 (50.2%) were randomized to the liberal arm. A total of 564 participants (20.6%) experienced at least 1 major septic complication within 30 days of surgery (sepsis, 265 [9.7%]; SSI, 409 [14.9%]; anastomotic leak, 78 [2.8%]; pneumonia, 104 [3.8%]); 22 participants (0.7%) died within 30 days. A total of 440 participants (16.4%) either died or had persistent disability within 90 days after surgery; 46 participants (1.7%) developed pulmonary edema, 157 (5.9%) developed acute kidney injury, and 285 (10.4%) had unplanned ICU admissions within 30 days of surgery. Results from the RELIEF trial have been published elsewhere.^25^

**Table 1.** Descriptive Statistics of Adiposity Measures for 2755 Participants

Measure	Mean (SD)	Median (IQR)
Waist circumference, cm	107.2 (19.5)	106.7 (93.5-120.0)
WHR	0.97 (0.09)	0.97 (0.91-1.02)
BMI	31.4 (8.7)	29.8 (25.1-36.1)

Abbreviations: BMI, body mass index (calculated as weight in kilograms divided by height in meters squared); IQR, interquartile range; WHR, waist-to-hip ratio.

Despite the numerous nonlinear models assessed, the best-fitting model for all 6 binary outcome variables and the quality of recovery outcome variable was determined to be the model with a simple linear term for the adiposity variable. Figures displaying fitted risk predictions for each outcome and adiposity variable with 95% confidence bands are provided in eFigure 2 to eFigure 7 in Supplement 2. The comparative indices of value of each predictor for the binary outcomes are presented in Table 2. The results for the outcomes were almost completely consistent in ordering of magnitude, ie, for all outcomes except unplanned ICU admission, waist circumference was observed to have the largest OR and more favorable AUC, NRI score, IDI score, and Brier score compared with other adiposity variables (eg, composite 30-day mortality or major septic complication, waist circumference: OR, 1.44; 95% CI, 1.28-1.62; *P* < .001; AUC, 0.641; NRI, 0.266; IDI × 10^4^ = 152.98; Brier score, 0.162; WHR: OR, 1.15; 95% CI, 1.03-1.28; *P* = .01; AUC, 0.621; NRI, 0.199; IDI × 10^4^ = 28.47; Brier score, 0.164; BMI: OR, 1.33; 95% CI, 1.17-1.50; *P* < .001; AUC, 0.629; NRI, 0.205; IDI × 10^4^ = 85.61; Brier score, 0.163). It also had the largest adjusted OR in the models with all 3 adiposity variables included together (eg, composite 30-day mortality or major septic complication, waist circumference: OR, 1.58; 95% CI, 1.25-2.01; *P* < .001; WHR: OR, 0.96; 95% CI, 0.84-1.10; *P* = .55; BMI: OR, 0.90; 95% CI, 0.72-1.12; *P* = .32) (eTable 2 in Supplement 2). However, these differences were statistically significant only for the composite outcome of septic complications or death within 30 days of surgery, indicating that chance cannot be ruled out as the reason for the apparent greater value of waist circumference compared with other outcomes. There was no apparent favor for any adiposity measure for unplanned ICU admission and quality of recovery (Table 3).

**Table 2.** Results for 6 Binary Outcomes Among 2755 Participants

Measure	OR for Each SD Increase in the Adiposity Variable (95% CI)	*P* Value	AUC	NRI	IDI × 10^4^	Brier Score
**Composite 30-d Mortality or Major Septic Complication, 2636 Participants**						
Waist circumference	1.44 (1.28-1.62)^a^^,^^b^	<.001	0.641^b^^,^^c^	0.266	152.98^a^^,^^b^	0.162^a^^,^^b^
WHR	1.15 (1.03-1.28)	.01	0.621	0.199	28.47	0.164
BMI	1.33 (1.17-1.50)	<.001	0.629	0.205	85.61	0.163
**Composite Disability Event or Death, 2661 Participants**						
Waist circumference	1.27 (1.12-1.45)	<.001	0.690	0.139	55.88	0.129
WHR	1.12 (1.00-1.26)	.04	0.684	0.118	15.88	0.129
BMI	1.19 (1.04-1.36)	.01	0.684	0.118	27.29	0.129
**Major Septic Complications, 2636 Participants**						
Waist circumference	1.45 (1.29-1.63)^a^^,^^b^	<.001	0.641^b^^,^^c^	0.270	157.37^a^^,^^b^	0.160^a^^,^^b^
WHR	1.16 (1.04-1.29)	.01	0.621	0.209	31.82	0.163
BMI	1.33 (1.18-1.51)	<.001	0.629	0.212	89.24	0.162
**Pulmonary Edema, 1695 Participants**						
Waist circumference	1.75 (1.17-2.61)	.01	0.840	0.371	123.58	0.024
WHR	1.27 (0.96-1.67)	.09	0.833	0.202	60.47	0.024
BMI	1.65 (1.07-2.55)	.02	0.838	0.250	95.54	0.024
**Acute Kidney Injury, 2598 Participants**						
Waist circumference	1.36 (1.11-1.67)	.003	0.716	0.232	45.67	0.055
WHR	1.29 (1.08-1.53)	.005	0.712	0.138	39.99	0.055
BMI	1.19 (0.95-1.48)	.13	0.709	0.080	13.98	0.055
**Unplanned ICU Admission, 2661 Participants**						
Waist circumference	1.09 (0.93-1.27)	.31	0.658	0.078	4.01	0.093
WHR	1.14 (0.99-1.31)	.07	0.659	0.143	12.85	0.093
BMI	0.98 (0.83-1.16)	.83	0.657	0.000	0.68	0.093

Abbreviations: AUC, area under the receiver operating characteristic curve; BMI, body mass index; ICU, intensive care unit; IDI, integrated discrimination improvement; NRI, net reclassification improvement; OR, odds ratio; WHR, waist-to-hip ratio.

^a^ Waist circumference vs WHR, *P* < .01.

^b^ Waist circumference vs BMI, *P* < .05.

^c^ Waist circumference vs WHR, .01 < *P* < .05.

**Table 3.** Results From the Regression Models for the Association of Adiposity Variables With Quality-of-Recovery 15 Scores for 2668 Participants

Variable	Univariate Model			Multivariate Model^a^		
	Coefficient^b^	*P* Value	*R*^2^, %		Coefficient^b^	*P* Value
Waist circumference	−0.036	.13	7.8		−0.052	.28
WHR	−0.040	.07	7.8		−0.024	.36
BMI	−0.014	.58	7.7		0.034	.45

Abbreviations: BMI, body mass index; WHR, waist-to-hip ratio.

^a^ *R*^2^ from model with all 3 adiposity variables together is 7.8%.

^b^ Standardized regression coefficient.

## Discussion

Our multicenter study compared the predictive value of 3 different obesity metrics, ie, BMI, waist circumference, and WHR, for postoperative complications in a cohort of 2755 patients following major abdominal surgery. We observed that waist circumference possessed the greatest value across a range of indices, as follows: association (OR), discrimination (AUC, NRI score, and IDI score), and smallest prediction error (Brier score). Although these differences appear small in magnitude (Table 2), they were beyond explanation by chance using our bootstrap procedure for obtaining *P* values.

There can be large variation in visceral adiposity at any given BMI.^26^ Our analyses, which adjusted the adiposity variables for each other, indicated that the association of waist circumference with all outcomes besides unplanned ICU admission and quality of recovery was largely unchanged after adjustment for BMI and WHR (eTable 2 in Supplement 2).

Our study provides robust evidence and adds valuable information to the existing literature on waist circumference. Consistent with our findings, a study on 152 patients who underwent rectal cancer surgery^15^ reported waist circumference to have a stronger association with superficial infections and 1 or more postoperative complications than BMI. Each increase of 10 centimeters in waist circumference was found to increase the odds of infection by 98%.^15^ Another large prospective study on elective colorectal surgery^13^ found that BMI was associated with increased risk only for abdominal wall complications. They observed that WHR was associated with intraoperative complications, conversion to an open operation, medical postoperative complications, and reoperations, whereas waist circumference was associated with surgical postoperative complications.^13^ A significant association of waist circumference with postoperative adverse outcomes, independent of BMI, has also been reported in 7446 patients who underwent coronary artery bypass.^27^ Several previous studies on patients who underwent laparoscopic colorectal surgery^28,29,30^ have reported visceral fat area, quantified using preoperative computerized tomographic images, to have stronger associations with technical difficulty, surgical time, and postoperative complications than BMI. However, these studies were conducted on small samples in the Asian population, limiting their generalizability.

It is said that SSI is the most common hospital-acquired infection, occurring in up to 20% of patients^31^; SSI is associated with increased length of hospital stay, reoperation rates, readmission rates, and up to an 11-fold increased risk of mortality.^32,33^ Numerous theories have been proposed to explain the higher risk of septic complications in individuals with obesity. These include prolonged operative time,^34^ reduced subcutaneous tissue oxygenation resulting in relative hypoxia in wounds, impairment in collagen synthesis and immune system functioning,^35^ and altered pharmacokinetics in individuals with obesity requiring antibiotic dose adjustments.^36^ Waist circumference is a marker of abdominal obesity and is associated with a high concentration of inflammatory mediators, such as interleukin 6, C-reactive protein, and tumor necrosis factor α, which are associated with insulin resistance and inflammation.^37,38^ Reduced vascularity in the abdominal adipose tissue has been proposed as a reason for the better predictive ability of waist circumference compared with BMI.^15^

Major surgery has been shown to be associated with reduced quality of life 30 days after the procedure.^39^ A significant negative association has been reported between postoperative complications and the well-being of patients who underwent surgery.^40^ Occurrence of SSI can adversely impact patients’ quality of life^41^ and leave patients frustrated and dissatisfied with their health care.^42^ A low health-related quality of life immediately following surgery was shown to be associated with long-term low quality of life.^43^ In fact, surgical complications are associated with a patient’s quality of recovery even up to a year after their surgery.^40^ However, a 30-day assessment of patients’ health-related quality of life is likely to be a more accurate reflection of their perception of perioperative management and in-hospital care.^39^ In our study, we observed a negative association of waist circumference with quality of recovery, which was larger than the associations for BMI and WHR. However, this evidence was not conclusive.

### Strengths and Limitations

The strengths of our study include its use of data from a large, international, multicenter randomized clinical trial among patients undergoing a wide range of major abdominal surgical procedures. End points were strictly determined, including the occurrence of 30-day septic complications confirmed by an end point adjudication committee based on source documentation. Outcome ascertainment and the results of our study are therefore easily generalizable. Further, to our knowledge, this is the first large-scale study to assess the value of adiposity measures in assessing risk of patient-centered end points, namely postoperative disability and quality of life.

Limitations of our study included that we assessed practical, bedside surrogate measures of body fat and did not use radiologic methods to confirm the degree of adiposity. Measurement of waist circumference and WHR can vary according to the protocol followed and be subject to errors. However, in a systematic review, Ross et al^44^ found that variation in the waist circumference measurement protocol did not influence its risk predictive ability. In addition, reproducibility of waist circumference measurements has been found to be high even when 4 common anatomic sites were used.^45^ Although waist circumference and WHR are considered crude estimates of adiposity, a previous study on 789 individuals^46^ concluded that waist circumference was the best measure of abdominal visceral adiposity assessed by computed tomography compared with BMI, body fat percentage, and WHR. Our study did not involve an assessment of metabolic syndrome or cardiorespiratory fitness because we only intended to compare the 3 anthropometric indices. Previous studies on abdominal surgery have shown higher rates of superficial and deep wound SSIs but not organ or space SSIs among patients with higher BMI.^47,48^ Although our findings support an increased risk of SSI with obesity, we did not explore the types of SSI. Waist circumference was observed to have the highest OR and largest AUC; however, the absolute differences over other adiposity measures were small. In addition, the reduced variance of BMI in our study population may have inhibited detection of associations that may be observed in populations with greater variance. We recommend further large-scale prospective studies to investigate the association of adiposity measures with other individual adverse outcomes.

## Conclusions

In this secondary analysis of the RELIEF randomized clinical trial, waist circumference was observed to be superior to other adiposity indices in predicting 30-day major septic complications alone or in conjunction with death following elective major abdominal surgery. Our findings suggest that waist circumference is a useful adiposity measure that should be incorporated in preoperative risk assessment for such complications.

## Notes

Supplement 1. **Trial Protocol**

Supplement 2. **eTable 1.** Participants’ Eligibility Criteria **eTable 2.** Results From Regression Analyses for All Outcomes With Adjusted Adiposity Variables **eFigure 1.** Histograms of Waist Circumference, Waist-to-Hip Ratio, and BMI for 2755 Participants **eFigure 2.** Fitted Risk Prediction Model for Disability or Death to 90 Days **eFigure 3.** Fitted Risk Prediction Model for Septic Complications or Death to 30 Days **eFigure 4.** Fitted Risk Prediction Model for Any Major Septic Complication **eFigure 5.** Fitted Risk Prediction Model for Pulmonary Edema **eFigure 6.** Fitted Risk Prediction Model for Acute Kidney Injury **eFigure 7.** Fitted Risk Prediction Model for Unplanned ICU Admission to 30 Days
