# Supplementary material for: Association of Obesity With Septic Complications After Major Abdominal Surgery: A Secondary Analysis of the RELIEF Randomized Clinical Trial
Source: JAMA Netw Open. 2019 Nov 27;2(11):e1916345. doi: 10.1001/jamanetworkopen.2019.16345 (PMC6902846; doi:10.1001/jamanetworkopen.2019.16345)
Supplement: Supplement 1. — Trial Protocol [file jamanetwopen-2-e1916345-s001.pdf]

The RELIEF Trial

REstrictive versus LIbEral Fluid Therapy in Major Abdominal Surgery

www.relief.org.au

On behalf of the Australian and New Zealand College of Anaesthetists Trials Group (ANZCA TG), and the Australian and New Zealand Intensive Care Society Clinical Trials Group (ANZICS CTG)

ClinicalTrials.gov Identifier: NCT01424150

Protocol Version 2: 11th April 2013

Study title: Restrictive versus liberal fluid therapy in major abdominal surgery

Protocol Number: Anaesthesia Research 046

Sponsor: Alfred Health, Melbourne

Funding source: The Australian National Health and Medical Research Council (APP1043755)

AGREEMENT

This document is confidential. The Investigators declare that they have read the final study protocol and any amendments. The Investigators will conduct the study according to the procedures specified in the study protocol, and in accordance with ICH GCP notes for Guidance on Good Clinical Practice (CPMP/ICH/135/95) and the Australian NH&MRC National Statement on Ethical Conduct in Research Involving Humans.

....	....
Investigator	Date

RELIEF: REstrictive versus LIbEral Fluid Therapy in Major Abdominal Surgery

Principal Investigator
Paul Myles, MB.BS, MPH, MD, FCARCSI, FANZCA, FRCA
Director, Department of Anaesthesia and Perioperative Medicine, Alfred Hospital and Monash University

Intensive Care
Rinaldo Bellomo, MB.BS, MD, FRACP, FCICM, PGDipEcho
Co-Director of the ANZIC-RC and the Director of Intensive Care Research at Austin Health

Anaesthesia
Tomas Corcoran, MB, BCh, BAO, MRCPI, FCARCSCI, MD, FCICM
Department of Anaesthesia and Pain Medicine, Royal Perth Hospital; School of Medicine and Pharmacology, University of Western Australia

Philip Peyton, MD, PhD, MB.BS, FANZCA, PGDipEcho
Staff Anaesthetist, Department of Anaesthesia, Austin Health

David Story, MBBS, MD, BMedSci, FANZCA
Chair of Anaesthesia; and Head, Centre for Anaesthesia, Perioperative and Pain Medicine; The University of Melbourne

Kate Leslie, MB.BS, MD, M Epi, FANZCA
Staff Anaesthetist, Department of Anaesthesia and Pain Management, Royal Melbourne Hospital; University of Melbourne; Monash University

Surgery
Chris Christophi, MB.BS, FRACS
Director of Surgery, Austin Hospital and University of Melbourne

Jonathan Serpell, MB.BS, MD, Med, FRACS, FACS
Director, Department of General Surgery, Alfred Hospital and Monash University

Statistician
Andrew Forbes MSc, PhD
Head, Biostatistics Unit, Department of Epidemiology & Preventive Medicine, Monash University

Research Manager: Sophie Wallace

Data and Safety Monitoring Committee
Monty Mythen (Chair, *intensivist*, Smiths Medical Professor of Anaesthesia & Critical Care, University College London (UK); Russell Gruen (*surgeon*), Guy Ludbrook (*anaesthetist*), John McNeil (*epidemiologist*), Katherine Lee (*statistician*).

Endpoint Adjudication Committee: David McIlroy, Mark Shulman, Jamie Cooper

Governance and Data Quality Manager: Stephanie Poustie

ABBREVIATIONS

ACE – angiotensin converting enzyme
ANZCA TG – Australian and New Zealand College of Anaesthetists Trials Group

ANZICS CTG – Australian and New Zealand Intensive Care Society Clinical Trials Group
APTT – activated partial thromboplastin time
ARB – angiotensin-II receptor blocker
ASA – American Association of Anesthesiologists
ATACAS – Aspirin and Tranexamic Acid for Coronary Artery Surgery trial
BMI - Body Mass Index
BP – Blood Pressure
CXR – Chest X-Ray
CRF – Case Report Form
CT - computed tomography scan
CVP – Central Venous Pressure
CD - Compact Disc
CDC - Centers for Disease Control and Prevention
CRP - C-reactive protein
DO2 – Tissue oxygen delivery
DSMC – Data Safety and Monitoring Committee
EAC – Endpoint Adjudication Committee
ECG - Electrocardiography
ENIGMA – Evaluation of Nitrous Oxide in the Gas mixture for Anaesthesia
ENIGMA-II - Nitrous oxide anaesthesia and cardiac morbidity after major surgery
ERAS – Enhanced Recovery After Surgery
FiO2 – Fraction of Inspired Oxygen
FTc – Flow Time Corrected
GA – General Anaesthesia
GCP – Good Clinical Practice
GFR - Glomerular filtration Rate
HR – Heart Rate
HDU – High Dependency Unit
INR - International Normalized Ratio
ICP – Intracranial Pressure
ICU – Intensive Care Unit
IV - Intravenous
IVRS – Interacive Voice Response System
L - Litres
PRN - as the occasion arises; as needed.
RIFLE - Risk, Injury, Failure, Loss, and End-stage kidney classification
NHMRC – Australian National Health and Medical Research Council
NHSN - National Healthcare Safety Network
mmol/L – Millimole per Litre
mmHg - millimetres of mercury
OR – Operating Room
PA – Pulmonary Artery
PaO2 – Arterial partial pressure of oxygen
PI&CF – Patient Information and Consent form
PPV - Positive Pressure Ventilation
QoR-40 – 40-item Quality of Recovery score
SAFE - Saline versus Albumin Fluid Evaluation study
sBP – Systolic Blood Pressure
SVV – Stroke Volume Variation
TGA – Australian Therapeutic Goods Administration
TOE – transoesophageal echocardiography, or TEE
WCC – White Cell Count
WHODAS - World Health Organization Disability Assessment Schedule

<u>TRIAL SUMMARY</u>

Design: This will be a large, randomized, parallel-group, controlled trial. After stratification by centre *and* planned ICU/HDU admission (or not), patients will be randomly assigned from a computer-generated list (1:1) to either a Restrictive or Liberal fluid Group.

Group 1 = Restrictive fluid regimen (intraoperative and 1st 24 h ≈2.5 L)
Group 2 = Liberal fluid regimen (intraoperative and 1st 24 h ≈5.5 L)

Sample Size: 2800 patients

Study Duration: 3 years

Primary Endpoint
Disability-free survival up to 1 year: survival and freedom from new-onset disability, the latter being a persistent (>6 months) reduction in functional status as defined by a 25% (4-point) or greater increase in the 12-item version of WHODAS to a final score of at least 25%. Disability will be assessed by the participant, but if unable then we will use the proxy's report. The date of onset of new disability will be recorded.

Interim analysis (& DSMC review): at n= 1000 and 2000 patients

1. AIM OF THE TRIAL

To investigate the effectiveness of fluid restriction (vs. liberal), and the possible effect-modification of goal-directed therapy (eg. oesophageal Doppler, Flotrac®). The first will be randomly assigned; the latter will be measured covariates according to local practices and beliefs.

The optimal fluid regimen and haemodynamic (or other) targets for patients undergoing major surgery are based on rationales that are not supported by strong evidence. Practices vary substantially; guidelines are vague, small trials and meta-analyses are contradictory. The strongest and most consistent evidence, and biological plausability regarding tissue oedema, supports a restrictive fluid strategy. There is less (and more contradictory) evidence supporting goal-directed therapy using a flow-directed device and/or dopexamine, and use and choice of colloids. A large, definitive clinical trial evaluating perioperative fluid replacement in major surgery is required.

1.1 Study Hypotheses

A restrictive fluid regimen for adults undergoing major abdominal surgery leads to reduced complications and improved disability-free survival when compared with a liberal fluid regimen.

Secondary hypotheses: The effects of fluid restriction are similar whether or not goal-directed therapy is used (assessed as a statistical test of interaction). A restrictive fluid regimen will reduce a composite of 30-day septic complications and mortality.

2. BACKGROUND

Anaesthetists typically manage perioperative hypotension in the first instance with an intravenous (IV) fluid bolus of a balanced salt crystalloid solution, or sometimes with one of several colloids. If persistent or more profound hypotension occurs, particularly in the intraoperative period when anaesthetic drug-induced vasodilation is common, an IV vasoconstrictor (typically metaraminol bolus prn) is used. Similar approaches are used in the intensive care unit (ICU) and surgical wards. We simply don't know whether using a 'liberal' fluid strategy based primarily on supplemental IV fluids, or a 'restrictive' strategy based on altered haemodynamic goals and/or vasopressor drug therapy, is best for most patients undergoing major surgery. The evidence base for fluid management in the postoperative setting is poor and is insufficient to guide our practice (1-4). Anaesthetists, intensivists and surgeons differ in their approaches to perioperative fluid therapy (5, 6).

Around 250 million people undergo major surgery each year around the world (7), with about 2 million being in Australia (1 in 10 Australians), and a growing proportion (now 40%) being elderly. By 2056 in Australia, more than 8.5 million anaesthetics (>50%) will be administered to patients over the age of 65 (8). These patients and many others have co-existent medical diseases that add risk to the procedure. The personal, social and economic consequences of postoperative complications, additional hospital stay, and long-term disability, are great.

Both colloids and crystalloids are used for fluid resuscitation and maintenance, but it is the amount of fluids administered and the goals of resuscitation that need re-evaluation. Since the 1950s, when it was first claimed that after surgery fluids are redistributed to a theoretical 'third space' (9), perioperative IV fluid replacement has included replacement of such third-space losses with crystalloid. In fact there are many reasons why clinicians administer generous amounts of IV fluids during and after surgery. Concerns about reversing preoperative dehydration, support ingthe circulation after general and regional anaesthesia, avoiding gut hypoperfusion and promoting tissue oxygen delivery, avoiding blood transfusion, and maintaining urine output are common (10-12). Optimizing tissue perfusion typically requires more fluid than indicated by normal clinical criteria or with invasive monitoring (10). Occult hypovolaemia and intraoperative gut hypoperfusion occurs in around 60% of major surgery patients, both of which are linked to increases in morbidity and mortality (11). Further support for this comes from some studies showing that a liberal fluid strategy in patients undergoing minor surgery, mostly in the ambulatory setting, improves early recovery measures such as dizziness, nausea and thirst, and may improve pulmonary function, exercise capacity, and shorten hospital stay (13). Similarly in the ICU setting, with some small trials suggest that fluid supplementation and optimized haemodynamics reduce organ dysfunction, postoperative morbidity and death (14, 15).

If fluid administration is restricted it is likely that hypotension will be treated with vasopressor therapy. Vasopressors may impair organ perfusion, threaten local tissues at the site of IV administration, cause arrhythmias, or be mistakenly used when hypovolaemia is the underlying cause.

But excess fluid administration causes oedema, with increased pulmonary morbidity (16), impaired coagulation (17), bacterial translocation and sepsis (18), and poor wound healing (19). In contrast to the above, other small trials of patients undergoing abdominal surgery have found that fluid restriction lead to reduced morbidity and hospital stay (12, 13). This conflicting evidence explains why there are diverse and varied practices around the world. Several expert guideline/consensus statements have been published, with most supporting restrictive fluid administration (2, 20). But all come to similar conclusions: High-grade evidence regarding the optimal fluid regimen is currently lacking (20).

2.1 Liberal or Restrictive IV Fluid Resuscitation

Traditional perioperative IV fluid regimens in abdominal surgery can lead to patients receiving 3 to 7 L of fluid on the day of surgery and more than 3 L/day for the following 3 to 4 days, leading to a 3- to 6-kg weight gain (21, 22). Several small trials have compared restrictive and liberal fluid regimens (3, 23, 24).

Lobo et al (15) did a tightly-controlled randomized trial in 20 adult patients having colonic surgery. The liberal group, representing 'standard' care, received IV fluids in accordance with their present hospital practice (≥3 L/day) and the restrictive group received ≤2 L water and sodium 77 mmol per day. All patients had no comorbidity other than colonic cancer. The restrictive group had shorter median gastric emptying times, less complications (0 vs. 7, P=0.01) and shorter hospital stay (6 vs. 9 days, P=0.001). Brandstrup et al (17) did a randomized trial comparing similar fluid regimens in 172 colorectal surgical patients. The restrictive group had fewer postoperative complications (33% vs. 51%, P=0.013) and less deaths (0 vs. 4, P=0.12). Nisenavich et al (25) compared liberal and restrictive fluid regimens in 152 patients undergoing elective abdominal surgery. The restrictive group had faster return of bowel function, less complications (P=0.046), and shorter hospital stay (P=0.01). Similar benefits were found in recent trials in colorectal and abdominal aortic surgery (26, 27).

However, Kabon et al (26) compared similar fluid regimens in 253 colorectal surgical patients and found no difference in the rates of wound infection, restrictive group 14% vs. liberal group 11% (P=0.46). Holte et al (22) compared two fluid regimens with physiological recovery as the primary outcome measure in 32 patients undergoing fast-track colonic surgery. The rate of complications tended to be higher in the restrictive group (6 vs. 1, P = 0.08). A meta-analysis of the fluid trials up to 2007 (3) found restrictive regimens reduced overall complications, OR 0.41 (95% CI: 0.22-0.77), P=0.005; but the authors noted the heterogeneity of fluid regimens and definitions of outcomes. Another two recent small trials found either no benefit (27) or harm (28).

We have done an updated meta-analysis of relevant trials (12 trials, 1160 patients) to evaluate the overall effect of fluid restriction on mortality (see Fig 1) and some morbidities (23). We could not pool overall complications because of their variability and inconsistency of counting. About half the trials did not measure or report mortality, so this outcome is underpowered. We found some possible benefits of fluid restriction:

- Pneumonia: RR 0.43 (95% CI: 0.20-0.94); P=0.03
- Pulmonary oedema: RR 0.22 (95% CI: 0.06-0.78); P=0.02
- Hospital stay: restrictive groups 2 days less (95% CI: 0.5-3.4); P=0.009
- Hospital mortality: RR 0.59 (95% CI:0.2-2.0); P=0.40

Figure 1. Mortality

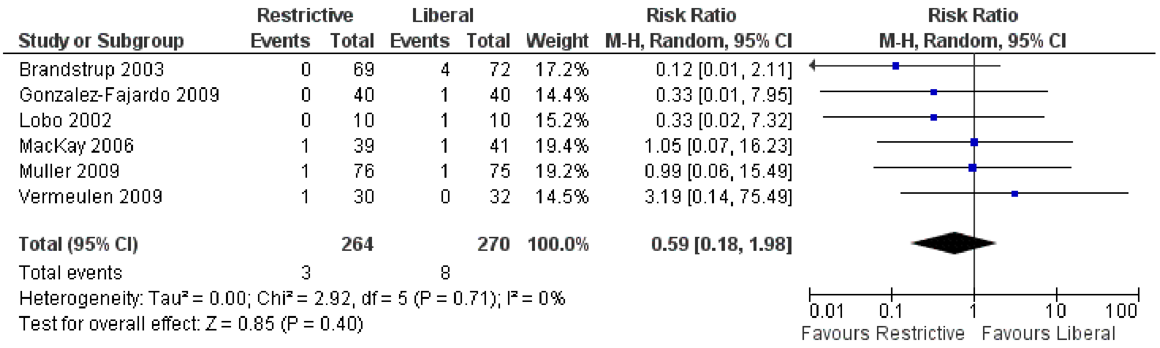

Our results show fluid restriction seems very promising and could lead to marked improvements in patient outcomes, but a large definitive trial is needed to generate the reliable evidence needed to change practice around the world.

An earlier meta-analysis that included less relevant trials (4) found that the range of ‘liberal’ IV fluid replacement varied from 2,750 to 5,388 ml compared with 998 to 2,740 ml in the ‘restrictive’ regimen. Like others (3) they noted that the fluid regimens and outcomes were inconsistently defined and only two studies reported perioperative care principles and discharge criteria. These and others have argued for a carefully designed trial that incorporates such details.

2.2 Crystalloid or Colloid Fluid Resuscitation?

Colloid proponents have argued that colloids lessen the risk of oedema because of the higher oncotic pressure, and textbooks typically recommend a 3-5 fold ratio of crystalloid to colloid volumes for acute fluid resuscitation. But the oncotic pressure effect may be lost if colloids leak and remain in the interstitial spaces. This perhaps explains why recent large trials have found that CVP and pulmonary function are comparable with both crystalloids and colloids (31-33). The SAFE study found that the volume of crystalloid needed for resuscitation at 24 h was only 1.3-fold larger than that of 4% albumin (29). There is concern regarding the safety of colloids (30-33).

The weight of evidence downplays the superiority of any particular IV fluid (crystalloid or colloid (29), type of colloid (3), or type of crystalloid. The main unresolved question is how much fluid to use, and whether haemodynamic- or flow-directed goals provide further benefit. However, in view of emerging evidence suggesting adverse effects of starch-based colloid solutions (30, 31), we recommend they NOT be used in this study.

2.3 Goal-directed Therapy: fluids and/or inotropes

CVP is an unreliable measure of intravascular status (32), but remains the most common monitor used to guide fluid resuscitation and vasopressor support. Relatively noninvasive monitors such as oesophageal Doppler and pulse contour analysis are becoming popular for intraoperative and ICU use (33), and there have been several positive trials (34-37), meta-analysis (23, 38), and guidelines (39) supporting their use. The strongest evidence is for oesphageal Doppler (39) but the device is infrequently used in Australian practice at present. Goal-directed strategies focus on

fluid responsiveness and typically require additional IV fluid supplementation, usually giving an extra 800 ml per case, and more postoperatively(23). These findings are hard to resolve when considering the apparent success of fluid restriction regimens described above.

One influential trial of 'optimized' care in the UK (15) in which 138 high-risk patients undergoing major abdominal surgery were randomly assigned to one of 3 groups: control, or 'pre-optimized' with either dopexamine or adrenaline. The control group remained on the general surgical ward with no preoperative fluid protocol. The intervention groups were admitted to the ICU for a minimum of 4 h before surgery, and had full haemodynamic monitoring including PA catheter. The two intervention groups were initially fluid optimized with colloid until pulmonary occlusion pressure 12 mm Hg was reached; red cell transfusion was used for haemoglobin <110 g/L. Patients then received inotrope therapy titrated to reach a target DO2 of 600 ml/min/m2 for up to 12±24 h after surgery. Hospital mortality in the protocol groups was 3%, compared with 17% in the control (P=0.007), and morbidity and hospital stay were significantly reduced in the dopexamine group. Interpretation of this study is difficult. It could be said that closer (and more expert) care in the ICU, compared with junior doctor-based ward care, was a key factor. Whether the target DO2 itself, inotrope therapy, additional fluids, or the combination of these factors is important is unclear. Two subsequent meta-analyses of dopexamine in major surgery had conflicting findings (42, 43), and a recent trial using FloTrac-guided fluid supplementation found(40) no effect on complication rate (40).

The most recent meta-analysis (41) of 29 trials involved 4805 patients found pre-emptive perioperative haemodynamic intervention significantly reduced mortality, OR 0.48 [95% CI:0.33–0.78]; P<0.0002; and surgical complications, OR 0.43 [0.34–0.53]; P< 0.0001. That is, supplemental fluids seem to improve outcome. Sub-group analyses showed similar effects with each type of intervention, including use of supplemental IV fluids alone:

Table 3. Subgroup Analysis for Number of Patients with Complications

Subgroup	No. of studies	No. of patients	No. of patients with complications in control group	Odds ratio (95% CI)
Monitor				
ODM	9	987	163/469 (35%)	0.41 (0.30–0.57)*
PAFC	10	1085	108/537 (20%)	0.54 (0.33–0.88)*
Other[a]	4	320	76/158 (48%)	0.32 (0.19–0.54)*
Therapy				
Fluids	9	742	126/372 (34%)	0.38 (0.26–0.55)*
Fluids and inotropes	14	1650	221/792 (28%)	0.47 (0.35–0.64)*
Goals				
CI/Do_2	12	982	169/461 (37%)	0.52 (0.37–0.74)*
FTc/SV	8	849	135/423 (32%)	0.41 (0.28–0.58)*
Other[b]	3	561	43/280 (15%)	0.26 (0.13–0.52)*
Resuscitation target				
Supranormal	6	469	133/227 (59%)	0.42 (0.29–0.63)*
Normal	17	1923	214/937 (23%)	0.43 (0.31–0.60)*

A later trial in 179 patients found no outcome benefit of goal-directed therapy, and possibly longer hospital stay (42).

One of the reasons for the varied results is that the focus should not be on the amount of IV fluid, but the timing and individualisation of such therapy. There may be an optimal amount, probably better targeted using a goal-directed approach (43).

2.4 "Fast-track" or "enhanced recovery from surgery" (ERAS) programs

There is a growing interest in facilitating recovery and earlier hospital discharge after colorectal and other abdominal surgery (43-45). ERAS programs typically include avoidance of bowel preparation, nasogastric and drain tubes; non-opioid analgesia; and promoting early postoperative mobilization and oral nutrition. A randomized trial comparing an ERAS program with traditional care in 156 patients undergoing colorectal surgery was stopped early because of apparent benefit (44), with less complications (21% vs. 50%, P=0.001) and a shorter hospital stay (5 vs. 9 days, P<0.001). A regression analysis revealed excess IV fluids (OR 4.2 [95% CI 1.7–10]; P=0.002) as an independent predictor of postoperative complications. A recent meta-analysis of ERAS studies has similarly found a significant reduction in complications and hospital stay (44). Most of the above fluid trials did not employ ERAS principles (4), and so we plan to include these in our study.

2.5 Measuring Outcome after Major Abdominal Surgery?

Most of the above-quoted studies pooled a variety of postoperative adverse outcomes into a single composite outcome ("complications"), for which there was often an imbalance in severity and duration, and with questionable long-term relevance to patients. Composite outcomes can be valid and important but only if properly constructed (45). Of course a hard endpoint after surgery is survival, but none of the above studies was sufficiently powered to detect a clinically important difference. Mortality is low after most types of surgery (48, 51) and so is an unattractive primary endpoint on which to base a sample size calculation.

It is unclear which of many adverse postoperative outcomes dominates any other. There is a strong argument to use patient-centred outcome measures. Quality of life is often used, but these instruments were not designed to be responsive after major surgery. Our 40-item quality of recovery score (QoR-40) has undergone psychometric evaluation, including utility and responsiveness testing (46, 47), and has been externally validated and used in many perioperative studies (52-54). But the QoR-40 is designed to measure outcome up to 30 days after surgery. Survival, and avoiding long-term disability, are likely to be the most important and highly valued outcomes for patients undergoing major surgery (55, 56). We thus plan to measure disability-free survival up to 1 year after surgery in this study.

Interim Long-term Outcome Data for ENIGMA-II and ATACAS trials: Our experience to date with 1-year follow-up for death/disability (using Katz ADLs) in our two current large international trials across >30 sites (48, 49) has had excellent follow-up, with <1% missing data (24 of 2,570 patients). For noncardiac surgery (n=1800) there have been 242 deaths and 286 with new disability (a combined rate of 31%). This event rate, from a lower risk study population, exceeds our assumptions used in our sample size calculation. Clearly, disability should not be ignored in perioperative outcome trials, and its inclusion can enhance study power.

2.6 Feasibility: Pilot Study

To ascertain current practices and support for this trial, we surveyed all members of both ANZCA and ANZICS Trials Groups (n=238) and found that >90% were comfortable with the proposed Group fluid regimens and were interested in participating in the trial (50).

We undertook a feasibility pilot study of the proposed trial at 3 centres. After ethics approval and patient consent, and surgeon, anaesthetist and intensivist support, we have demonstrated that we can successfully implement the fluid regimens both intraoperatively and postoperatively:

variable	Restrictive (n=41)	Liberal (n=41)	P value
Age, y	65 ± 12	67 ± 12	-
IV fluid (crystalloid + colloid)			
Intraoperative	1746 ± 748	2730 ± 1309	<0.0005
Total at 24 h postoperative	3167 ± 1625	5133 ± 2138	<0.0005
Postoperative			
Haemoglobin, g/L	110 ± 18	101 ± 17	0.014
Albumin, g/L	31 ± 6.7	27 ± 7.0	0.030
CRP, mg/L	108 ± 80	128 ± 75	0.33
Quality of recovery score	159 ± 20	154 ± 26	0.34
Median ICU stay, h	0 (0-15)	0 (0-19)	0.86
Median Hospital stay, days	8.1 (5.6-14)	8.4 (6.9-16)	0.30

To date there is no evidence of any adverse haemodynamic or renal effects with restrictive therapy (51).

In addition, we are currently undertaking a cohort study of 400 patients undergoing a range of elective surgeries to accurately measure and define rates of comorbidity, wellbeing and disability at 1, 3, 6, and 12 months after surgery. This will validate our follow-up and disability measurement techniques.

3. STUDY DESIGN

3.1 Experimental design

Large, multicentre, randomized, single blind, pragmatic trial, with patients randomly assigned to either Restrictive or Liberal fluid, stratified by site and planned HDU/ICU admission.

This is an effectiveness trial (61, 62) – some elements of the trial are deliberately left to the anaesthetist's discretion in order to reflect usual practice and maximise generalisability.

3.2 Subject Selection

3.2.1 Definition of Disease State

We are targeting patients undergoing planned major abdominal or pelvic surgery that includes a skin incision and operative duration expected to exceed two hours.

3.2.2 Source and Number

We will use similar procedures to those used by us successfully in previous multicentre studies. Simple eligibility criteria, and research nurse-screening and enrolment, ensure that recruitment is maximized.

2800 patients in total will be required for this study (1400 in each group).

3.2.3 Entrance Criteria

Inclusion criteria:

1. Adults (≥18 years) undergoing elective major surgery and providing informed consent
2. All types of open or lap-assisted abdominal or pelvic surgery with an expected duration of at least 2 hours, and an expected hospital stay of at least 3 days (for example, oesophagectomy, gastrectomy, pancreatectomy, colectomy, aortic or aorto-femoral vascular surgery, nephrectomy, cystectomy, open prostatectomy, radical hysterectomy, and abdominal incisional hernia repair)
3. At increased risk of postoperative complications, defined as at least one of the following criteria:
   - **a)** age ≥70 years
   - **b)** known or documented history of coronary artery disease
   - **c)** known or documented history of heart failure
   - **d)** diabetes currently treated with an oral hypoglycaemic agent and/or insulin
   - **e)** preoperative serum creatinine >200 μmol/L (>2.8 mg/dl)
   - **f)** morbid obesity (BMI ≥35 kg/m^2)
   - **g)** preoperative serum albumin <30 g/L
   - **h)** anaerobic threshold (if done) <12 mL/kg/min
   - **i)** or two or more of the following risk factors:
     - ASA 3 or 4
     - chronic respiratory disease
     - obesity (BMI 30-35 kg/m^2)
     - aortic or peripheral vascular disease
     - preoperative haemoglobin <100 g/L
     - preoperative serum creatinine 150-199 μmol/L (>1.7 mg/dl)
     - anaerobic threshold (if done) 12-14 mL/kg/min

Exclusion criteria:

1. Urgent or time-critical surgery
2. ASA physical status 5 – such patients are not expected to survive with or without surgery, and their underlying illness is expected to have an overwhelming effect on outome (irrespective of fluid therapy)
3. Chronic renal failure requiring dialysis
4. Pulmonary or cardiac surgery – different pathophysiology, and thoracic surgery typically have strict fluid restrictions
5. Liver resection – most units have strict fluid/CVP limits in place and won't allow randomisation
6. Minor or intermediate surgery, such as laparoscopic cholecystectomy, transurethral resection of the prostate, inguinal hernia repair, splenectomy, closure of colostomy – each of these are typically "minor" surgery with minimal IV fluid requirements, generally low rates of complications and mostly very good survival.

3.3 Study Procedures

3.3.1 General Description

Study Flow Chart

	Visit 1	Visit 2	Visit 3	Visit 4	Visit 5	Visit 6	Visit 7	Visit 8	Visit 9
	Preadmission Clinic/ preoperative visit	Day of Surgery	Post op day 1	Post op day 3	Day of discharge	30 day follow up phone call	3 month phone follow-up	6 month phone follow-up	12 month phone follow-up
Entry Criteria	x								
Informed Consent	x or x								
Demographics, Medical History	x or x								
ECG	x or x		x	if chest pain or elevated troponin					
Randomisation		x							
Blood tests Electrolytes	x or x		x	x					
Liver function tests	If clinically indicated		If clinically indicated						
HbA1C	Recommended in ALL diabetics								
CRP				X					
Blood tests Troponin Lactate		If clinically indicated							
IV fluids		x	x	x					
Web-based data entry		x			x	x	x	x	x
Wound inspection			If change of dressing	x	x	Medical record review			
QoR-15			x	x		x			
WHODAS	x					x	x	x	x
Adverse Events		x	x	x		x			

**Outcomes**						x			481
**Blood products**		x	x	x	x				

All procedures are based on successful strategies used in each of our previous large multicentre trials. Ethics Committee approval and informed consent will be obtained at all study centres. After enrolment, on the day of surgery, patients will be randomly assigned (1:1) to groups via either (both established) 24-hr freecall telephone or web-based service using a computer-generated code. All other perioperative clinical care will be according to standard practice. All relevant factors will be recorded on a trial case report form (CRF).

3.3.2 Perioperative Management

Preoperative period

ERAS perioperative care principles will be emphasized. All patients will receive prophylactic antibiotics according to established guidelines. Medications will be continued perioperatively unless or at the clinicians discretion, but we will recommend withholding ACE-inhibitors and ARBs on the day of surgery. We will record preoperative use of bowel preparation, fasting times, ERAS data, medications, and biochemistry and haematology results on the CRF.

Intraoperative period

Choice of anaesthetic agents and perioperative analgesia will be left to the discretion of the anaesthetist; such data will be recorded. We will emphasize the need to avoid hypothermia (<36 ^{o}C). Epidural use will be recorded as this may increase the risk of hypotension and need for IV fluids (63, 64), but such effects are likely to be small (52). We will record usage of all "advanced" monitoring devices (CVP, pulse contour analysis, TOE, oesophageal Doppler).

The acceptable limits of low BP, and a definition of 'hypotension', vary widely (66), though such a definition will be modified by older age, pre-existing hypertension, and cerebrovascular disease. We will use a general guideline of systolic BP <90 mmHg for more than 5 mins, but also ask the attending anaesthetist to modify their acceptable lower limit of sBP at the commencement of surgery, and, according to randomly-assigned group, treat hypotension with additional IV fluid or vasopressor therapy (see below). For example, in younger patients or those with pre-existing low BP it may be acceptable to tolerate a sBP of 85-95 mmHg, but in older patients, particularly those with pre-existing hypertension, a higher lower limit may be required. Such modification to the acceptable lower sBP will be recorded. For patients managed in a high dependency or ICU environment after surgery, hypotension will be similarly treated for the first 24 h after surgery.

Postoperative period

Patients will be followed daily and outcomes will be recorded until discharge. We will recommend that antihypertensive medications should be withheld until sBP is consistently at or above preoperative levels. Serum electrolytes, haemoglobin/haematocrit, and a 12-lead ECG will be ordered preoperatively and on day 1 after surgery. CRP will be measured on postoperative Day 3 and whenever sepsis is suspected (67, 68). Additional laboratory tests will be ordered if clinically indicated. On day 3 all patients will complete the 15-item quality of recovery score (QoR-15). On day 30 all patients will be contacted by phone to ascertain if they have experienced any outcomes, and if detected, further testing will be arranged. Documentation for such events will be sought in the hospital medical record and doctor's records. The QoR-15 will be repeated on day 30 along with WHODAS, and the WHODAS will be repated at 3-, 6- and 12-month follow-up to ascertain survival status and new-onset disability.

3.3.3 Clinical Observations

3.3.3.1 Primary Endpoint

Disability-free survival up to 1 year: survival and freedom from new-onset disability, the latter being a persistent (>6 months) reduction in functional status as defined by a 25% (4-point) or greater increase in the 12-item version of WHODAS to a final score of at least 25% (69, 70). Disability will be assessed by the participant, but if unable then we will use the proxy's report. The date of onset of new disability will be recorded. Further details are provided in the Procedures Manual.

3.3.3.2 Secondary Endpoints

Secondary endpoints include an *a priori* composite of 30-day mortality or major septic complications (sepsis, surgical site infection, anastomotic leak (53), and pneumonia), plus each individually, serum lactate (at 6 and 24 h), CRP (Day 3), pulmonary oedema, blood transfusion, acute kidney injury, ICU and hospital stay, unplanned re-operation, unplanned admission to ICU, and quality of recovery (QoR-15). We will use the following definitions:

1. *Death*: all-cause mortality at 90 days, then up to 12 months after surgery
2. *Death or severe disability* (WHODAS score ≥40) at 12 months after surgery
3. *Sepsis:* using Centers for Disease Control and Prevention (CDC) with National Healthcare Safety Network (NHSN) criteria (54): - SIRS plus infection (positive blood culture or purulence from any site)

4. *Surgical site infection*: if associated with purulent discharge and/or a positive microbial culture
5. *Pneumonia:* typical x-ray appearance and ≥2 of (i) temperature ≥38 ^{o}C, (ii) WCC >12,000, and (iii) positive sputum culture
6. *Acute kidney injury*: defined by RIFLE criteria, but <u>not</u> urine output – at least 2-fold increase in creatinine, or GFR decrease >50% (55); plus renal replacement therapy up to 90 days after surgery
7. *Pulmonary oedema*: respiratory distress or impaired oxygenation AND radiological evidence of pulmonary oedema
8. *Duration of mechanical ventilation:* additive for all episodes up to 90 days after surgery
9. *Total ICU stay:* including initial ICU admission and readmission times
10. *Hospital stay*: from the start (date, time) of surgery until actual hospital discharge
11. *Quality of recovery:* QoR-15 score (52, 73) on days 1, 3, and 30.

Fluid Therapy and Blood Transfusion: General Guidelines

Excessive fluid resuscitation can cause haemodilution (56) and dilutional coagulopathy, and this may increase the need for red cell and other blood transfusion (29). Blood transfusion is, of itself, associated with increased rates of sepsis and other postoperative complications (24, 25). All patients will have the same red cell transfusion trigger of 70 g/L, but this can be modified after assessment of cardiovascular risk (57, 58) or concern for active bleeding. Normal Saline, containing 154 mmol of sodium and 154 mmol of chloride per litre, is non-physiological and can lead to hyperchloraemic acidosis (59) and perhaps poorer outcome (60, 61). We will use a balanced salt solution as the routine fluid therapy in this study. The questionable value of urine output as a measure of kidney or other tissue perfusion will be emphasized (62).

Our study Group fluid regimens are aimed at distinct volume differences and according to recent recommendations (4, 69). The group-assigned fluid regimens will continue for at least 24 hours after surgery, or until cessation of IV fluid therapy (whichever occurs first). If the patient's clinical condition warrants modification to the type or rate of fluid administration, then such modifications can be made immediately. This does NOT imply that the patient is removed from the trial because we will analyze according to the intention-to-treat principle, but we will collect such data for secondary per-protocol and sensitivity analyses.

Management of Oliguria

It is a normal response of the body to attempt to conserve fluid in times of physiological stress. Oliguria (low urine output) is part of this homeostatic mechanism; there is no evidence it is harmful in the short term (first 24-48 h after surgery is common and not abnormal) (62). Nor is there any evidence that diuretics protect against AKI (63). We will however provide guidance to ward medical and nursing staff (see Procedures Manual).

4. Experimental control

4.1 Group assignment

This will be a large, randomized, parallel-group, controlled trial. After stratification by centre and planned ICU/HDU admission (or not), patients will be randomly assigned from a computer-generated list (1:1) to either a Restrictive or Liberal fluid Group.

A 24-hr interactive voice recognition system (IVRS) will be available. An alternative web-based randomisation service will also be available during the conduct of the trial.

This is an intention to treat trial. Any participant who is randomised will be followed for the duration of the trial (unless they withdraw consent) even if they are withdrawn from the active phase of the trial. Patients who do not complete the active phase of the study will not be replaced.

Liberal Protocol

The Liberal protocol group reflects common contemporary practices in Australia (31, 80)(76), and is consistent with previous international trials (21, 25, 78) – see Appendix. At the commencement of surgery a bolus of Hartmann's balanced salt or Ringer's lactate crystalloid 10 ml/kg followed by 8 ml/kg/h will be administered until the end of surgery – the latter can be further down-titrated after 4 hours if clinically indicated. <u>Important:</u> for the purposes of calculations of bolus and maintenance fluids in patients exceeding 100 kg, the maximal body weight will be set at 100 kg. A maintenance infusion will then continue at 1.5 ml/kg/h, for at least 24 hours, but this can be reduced <u>postoperatively</u> if there is evidence of fluid overload and no hypotension, and increased if there is evidence of hypovolaemia or hypotension. Alternative fluid types (crystalloid, dextrose, colloid) and electrolyte supplements will be allowed <u>postoperatively</u> in order to account for local preferences and patient biochemistry, for which we will collect data. For a 75-kg adult, the intraoperative volume (for a 4 h operation) will be 3150 ml (+colloid/blood replacement

for blood loss), and then around 2700 ml per day. That is, the first (intraoperative + postoperative) 24-h fluid administration will be about 5400 ml (P.T.O).

Restrictive Protocol

The Restrictive protocol group is designed to provide less than 2.0 L water and 120 mmol sodium per day. Induction of anaesthesia will be accompanied by an IV fluid bolus limited to ≤5 ml/kg; no other IV fluids will be used at the commencement of surgery (unless indicated by goal-directed device [see below]). Important: for the purposes of calculations of bolus and maintenance fluids in patients exceeding 100 kg, the maximal body weight will be set at 100 kg. Hartmann's balanced salt or Ringer's lactate crystalloid 5 ml/kg/h will be administered until the end of surgery, and bolus colloid/blood used intraoperatively to replace blood loss (ml for ml); then an infusion at 0.8 ml/kg/h until expedited cessation of IV fluid therapy within 24 hours. The rate of postoperative fluid replacement can be reduced if there is evidence of fluid overload and no hypotension, and can be increased if there is hypotension AND evidence of hypovolaemia. For a 75-kg patient and 4 h operation, intraoperative fluid volume will be 1875 ml (+colloid/blood replacement for blood loss). The first 24-h fluid administration will be around half that of the liberal group.

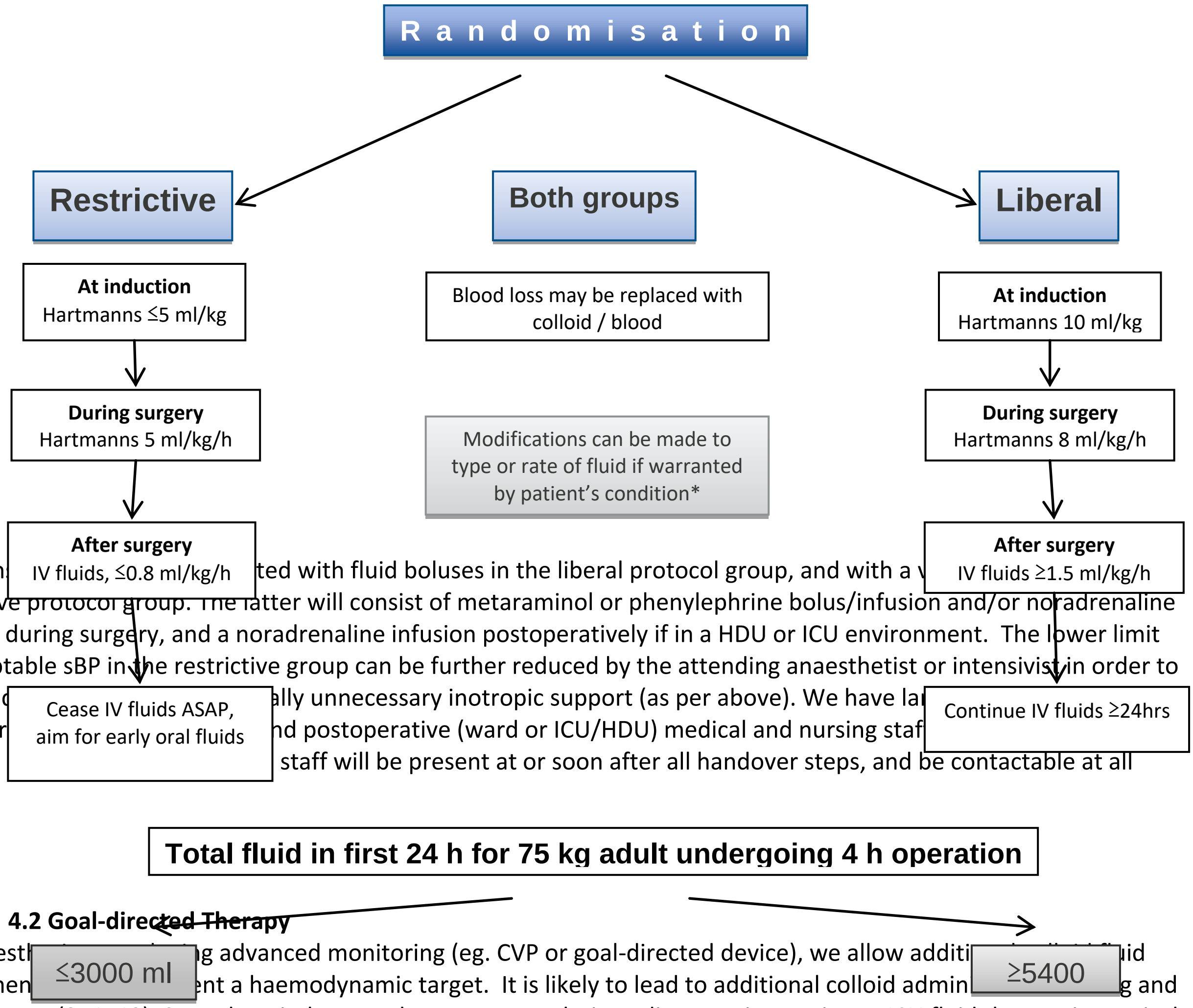

Hypoten[illegible]ted with fluid boluses in the liberal protocol group, and with a v[illegible] restrictive [illegible] group. The latter will consist of metaraminol or phenylephrine bolus/infusion and/or noradrenaline infusion during surgery, and a noradrenaline infusion postoperatively if in a HDU or ICU environment. The lower limit of acceptable sBP in the restrictive group can be further reduced by the attending anaesthetist or intensivist in order to limit flui[illegible]lly unnecessary inotropic support (as per above). We have la[illegible] flowchar[illegible]nd postoperative (ward or ICU/HDU) medical and nursing staf[illegible] patients [illegible] staff will be present at or soon after all handover steps, and be contactable at all hours.

4.2 Goal-directed Therapy

For anaesth[illegible]g advanced monitoring (eg. CVP or goal-directed device), we allow additi[illegible]id supplemen[illegible]ent a haemodynamic target. It is likely to lead to additional colloid admin[illegible]g and after surgery (3, 4, 40). Some hospitals use pulse contour analysis to direct perioperative or ICU fluid therapy in surgical patients, and some use oesophageal Doppler. Most rely upon conventional monitoring (HR, BP, urine output). We plan to test the effectiveness of each approach according to their local availability and use. The statistical analysis will focus on a test for interaction, to determine whether the effects of a fluid regimen work differently in those with and without any advanced monitoring. We anticipate that more than half will use (only) clinical measures.

For each of the goal-directed techniques, pulse/stroke volume variation or FTc will be measured before commencement of surgery and repeated at regular (say, 10-30 min) intervals intraoperatively. For those in the liberal protocol group, goal-directed therapy can continue postoperatively at 4 hourly intervals, for up to 24 hours after

surgery. If there is evidence of fluid responsiveness (eg. systolic pressure/volume variation of ≥13% (77)) at any of these times thenIV colloid or crystalloid 3-5 ml/kg can be given. Such data will be collected on the CRF.

**Colloid* (recommended) or crystalloid (3 ml/kg)**	**Liberal**	**Restrictive**
**Colloid/blood (using a transfusion threshold) bolus *if* acute bleeding**	**Yes**	**Yes**
***If* normotensive but monitoring suggests hypovolaemia (eg. low CVP or oliguria)**	**Yes**	**No**
***If* normotensive but goal-directed device suggests hypovolaemia (eg. FTc <0.33, ΔSV ≥10%, or SVV≥13%)**	**Yes**	**Consider**
***If* hypotensive (1) *and* hypovolaemia**	**Colloid***	**Colloid* (but limit) + vasoconstrictor**
**(2) *but not* hypovolaemic**	**Colloid* ± vasoactive**	**vasoactive therapy**

*** starch-based colloids are not recommended (30, 31)**

4.3 Blinding Procedure

Patients will be blinded to Group allocation. Anaesthetists, surgeons, and intensivists will have knowledge of Group identity. Similarly, it is expected that other surgical and nursing staff, and research staff conducting the in-hospital daily reviews, cannot be properly blinded to Group identity. But research staff conducting 1-12 mth follow-ups MUST be blinded to Group allocation.

4.4 Case Report Forms

For each form on which information is entered, the patient's initials, allocation number and the date of the visit must be entered in the appropriate space. The CRFs must be neatly handwritten with a black-ink ballpoint pen. Errors must be corrected by drawing a single line through the incorrect entry and writing in the new value positioned as close to the original as possible.

The correction must then be initialled and dated by the authorised individual making the change. Do not obliterate, write over, or erase the original entry when making a correction.
Case report forms should be opened as soon as possible following the start of screening and kept up to date as the patient continues the study.

As soon as possible after the end of each patient's participation in the study the CRF must be completed. All centres must store the paper based CRF according to GCP/ICP guidelines.

4.5 Web-based data entry

Following completion of the paper-based CRF, data will need to be entered by research staff to the database through a web-based data entry system. Further information can be found in the Procedures Manual. The system will audit the timeliness of data entry and reports will be generated the data monitoring committee regularly.

4.6 Data Base Production and Verification

Study data will be collected via the internet, monitored by the trial data management centre where all data fields are checked and automatically downloaded onto a database. At the end of the trial site-specific data will be sent to each site investigator on a CD, for long-term storage.

Study data will be collected in a paper based CRF, for transcription onto a web-database. We will maximize data quality and protocol standardization by arranging a start-up meeting at local scientific meetings or live streamed web based sessions, and will provide regular feedback to each centre via phone and the trial web-site, along with a monthly newsletter. A complete procedures manual will be produced. All study personnel will have 24-h access to the study coordinating centre to resolve any questions that arise. Further information can be found in the Procedures Manual.

4.7 Compliance Checks

Random audits of centres will be undertaken, to access the accuracy and legitimacy of the trial data. Statistical monitoring of the data completeness, data variance, and risk-appropriate endpoint rates will be done for all patient data.

4.8 Patient Completion/Withdrawal

All participants who are randomised will and undergo GA for surgery must be followed for the duration of the study (unless they withdraw consent) even if they are withdrawn from the active phase of the trial.

4.9 Repeat and Special Laboratory Tests

Serum electrolytes, haemoglobin/haematocrit, and a 12 lead ECG will be ordered preoperatively and if clinically indicated after surgery. All diabetics should have their HbA1C measured before surgery. Further tests will be ordered if clinically indicated.

4.10 Adverse Experiences

Serious adverse effects, serious adverse reactions, or suspected unexpected serious adverse reactions (SUSARs) are serious adverse events judged to be related to therapy.

At each visit/assessment, all adverse experiences either observed by the investigator or one of the clinical staff, or reported by the patient spontaneously or in response to a direct question will be evaluated by the investigator and noted in the adverse experience section of the patient's CRF. The nature of each experience, time of onset after surgery, duration, severity and relationship to treatment will be established. Any corrective treatment should be recorded on the appropriate pages of the CRF.

Adverse events should be documented at each assessment point throughout the study. Maximum intensity should be assigned to one of the following categories:

Mild - an adverse event which is easily tolerated by the patient, causing minimal discomfort and not interfering with everyday activities.

Moderate - an adverse event which is sufficiently discomforting to interfere with normal everyday activities.

Severe - an adverse event which is incapacitating and prevents normal everyday activities and/or requires therapeutic intervention (i.e. use of a prescription drug or hospitalisation).

Any serious adverse event should be reported by the local site investigator or research assistant within 24 hours by telephone or email to the local site investigator. Note that study endpoints do not need to be included as serious adverse events.

A preliminary telephone report should be followed by a full report which includes copies of relevant hospital case records, autopsy reports and other documents, where applicable.

A serious adverse experience is defined as any event which is fatal, life-threatening, permanently disabling or incapacitating or results in hospitalisation, prolongs a hospital stay or is associated with congenital abnormality, carcinoma or overdose.

Life threatening means that the patient was at immediate risk of death from the event as it occurred, ie. it does not include a reaction that, had it occurred in a more serous form, might have caused death. For example, drug induced hepatitis that resolved without evidence of hepatic failure would not be considered life threatening, even though drug induced hepatitis can be fatal.

Permanent disability means a permanent and substantial disruption of a patient's ability to carry out normal life functions.

More details for Adverse Event reporting will be found in the procedures manual.

5. BIAS CONTROL

This is a large trial, randomised with permuted blocks (by centre and ICU). Anaesthetists, surgeons, and intensivists will have knowledge of Group identity. Similarly, it is expected that other surgical and nursing staff, and research staff conducting the in-hospital daily reviews, cannot be properly blinded to Group identity. But research staff conducting Day 3, 1-12 mth follow-ups MUST be blinded to Group allocation. Secondary outcomes are clearly defined in the protocol; disputes will be resolved by blinded assessors (endpoint adjudication committee).

6. SAMPLE SIZE AND STATISTICAL ANALYSIS

All statistical analysis will be overseen by Prof Andrew Forbes, Monash University Department of Epidemiology and Preventive Medicine. The intention-to-treat population will include all patients randomly assigned to groups AND undergoing induction of anaesthesia.

Our sample size calculation is based primarily on our own data and other published studies. Our ENIGMA-II trial (n>5000 enrolled to date), with a lower risk study population, has a disability-free survival rate of 70% (15% mortality, 15% new disability) at 1 year after surgery). The most recent large data comes from the UK, where the 1-year mortality for open colorectal surgery was 17% in the 31,847 patients with pre-existing comorbidity (64). Reductions in serious complication rates have exceeded 25% in pooled analyses of similar studies (3, 76), and pre-existing major comorbidity increases mortality risk up to 16-fold (65). Using a type I error of 0.05 and survival analysis, with an expected one year disability-free survival probability of 65% (66) and a hazard ratio of ≥1.25, 1300 patients in each group will provide 90% power. Target recruitment will be set at 2800 patients to account for losses due to follow-up.

Analyses will be intention-to-treat. For analysis of the composite death-disability endpoint, we will use the Cox proportional hazards regression model; for secondary functional outcome (WHODAS), we will use ordinal logistic regression. Both analyses will be adjusted for age and ASA physical status. Incidence proportions for binary outcomes will be analyzed using chisquared tests, with covariate adjustment done using log-binomial regression. Results will be expressed with risk ratios and 95% CI. Other secondary endpoints will be compared with rank sum and/or t-tests as appropriate.

Planned sub-group analyses will assess patient sex, age groups, bowel surgery, and use of monitoring devices (including goal-directed techniques). For these we will undertake tests for interaction by adding terms to the regression models.

7. INTERIM ANALYSIS

Interim analyses will consider the defined study endpoints, but include a specific consideration of 90-day mortality (because the primary endpoint is not finalised until 1 year after study entry) after enrolment of 1000 and 2000 patients, adjusted according to the O'Brien and Fleming method. Results will be made available to the Data and Safety Monitoring Committee.

8. SECONDARY ANALYSIS

We plan several substudies (to be funded from other sources), each of which will have a separate protocol and authorship plan (using an expanded list of contributors). Additional blood tests and other investigations will be done at selected hospitals according to local interest and expertise.

- **8.1** Cost-effectiveness, to include hospital stay and complications as we have done previously (67)
- **8.2** Hyperchloraemic acidosis (to measure strong ion difference, Cl-, lactate, albumin)
- **8.3** Pulmonary oedema (to measure FiO2/PaO2 ratio, CT/CXR-confirmed atelectasis)
- **8.4** Coagulopaty (to measure blood loss, platelet count, fibrinogen, INR, APTT, Hb flux, transfusion)
- **8.5** Sepsis (to measure fever, WCC, CRP and possibly other biomarkers)
- **8.6** AKI and hepatic injury
- **8.7** Postoperative cognitive deficit
- **8.8** Feeding and return of bowel function
- **8.9** Wound healing and anastomotic leak
- **8.10** Late cancer recurrence.

9. PERSONNEL RESPONSIBILITIES

9.1 Investigators

The Steering Committee will consist of the principal investigator (PSM [Chair]), and other clinician-researchers in anaesthesia, surgery and intensive care medicine, plus the trial statistician – see below.

Each site investigator must ensure that all staff conducting the study are qualified to do so.

Each site investigator must submit the study protocol to the Ethics Committee or equivalent regulatory body and obtain approval prior to commencing the study.

Each site investigator must ensure that all staff involved with the study are fully instructed on the study procedures and are given access to the study protocol and other information relating to the study.

Each site investigator must ensure that the study is conducted in accordance with this protocol, ICH GCP notes for Guidance on Good Clinical Practice (CPMP/ICH/135/95) and in Australia with the NHMRC National Statement on Ethical Conduct in Research Involving Humans.

It is each site investigator's responsibility to ensure that written, informed consent is obtained from each patient prior to entering the study.

Each site investigator must ensure that the web-based CRFs are complete and accurate on completion of the study. Each site investigator will ensure that the quality control procedures are performed on both the CRFs and the data base.

It is the principal investigator's responsibility, in conjunction with the chief investigators, to write the Study Report at the completion of the study. Authorship guidelines are described in Section 11.

9.2 Monitor

Not applicable.

9.3 Sponsor

Alfred Health, as an investigator initiated study.

9.4 Steering Committee

The steering committee will include Paul Myles (chair), Rinaldo Bellomo, Tomas Corcoran, Chris Christophi, Andrew Forbes, Phil Peyton, David Story, Andrew Davies, Kate Leslie, Jonathan Serpell, and Sophie Wallace (trial manager)

9.5 Endpoint Adjudication Committee (EAC)

Confirmation reports of all detected outcomes will be de-identified and re-labelled with study number. The committee will consist of experienced perioperative physicians. Details are provided in the Procedures Manual. Their role will be to resolve any uncertainty as to any of the above outcomes: additional advice can be sought by consultation with sub-specialists.

10. DATA SAFETY MONITORING COMMITTEE

The committee consists of Prof Monty Mythen (Chair, *intensivist*, Smiths Medical Professor of Anaesthesia & Critical Care, University College London (UK); Co-Director, Surgical Outcomes Research Centre), Prof Russell Gruen (*surgeon*, Professor of Surgery and Public Health, The Alfred & Monash University Director, National Trauma Research Institute; Melbourne), Prof John McNeil (*epidemiologist and triallist*, Professor of Epidemiology and Preventive Medicine; Head, School of Applied Clinical and Public Health Sciences; Monash University), Prof Guy Ludbrook (*anaesthetist*, Professor of Anaesthesia, Flinders University), and Dr Katherine Lee (*independent statistician*, MCRI).

The DMSC will discuss the interim results and vote for continuation or stopping the trial. A majority vote to stop the trial will be communicated to the Steering Committee at the Trial Coordinating Centre according to predetermined stopping rules (as above) and consideration of other relevant evidence. Their conduct is to be guided by the paper by DeMets et al. (81). Further details are provided in the DSMC charter.

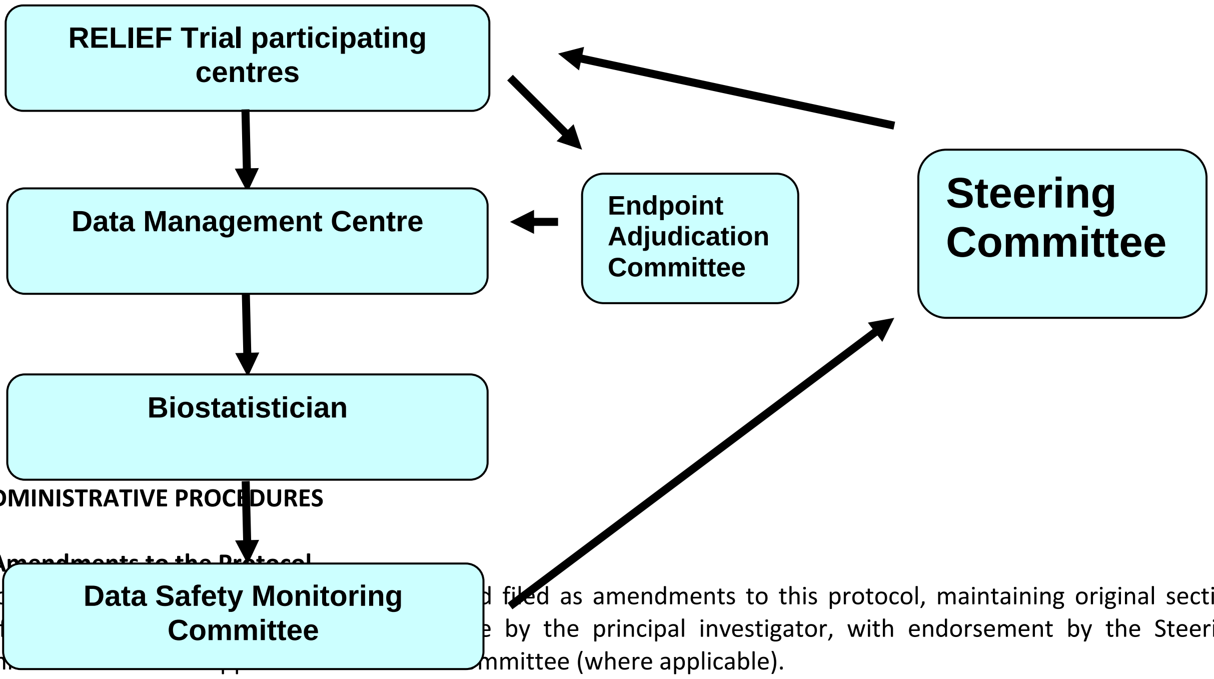

11. ADMINISTRATIVE PROCEDURES

11.1 A[illegible]

All mo[illegible]d filed as amendments to this protocol, maintaining original section identi[illegible]e by the principal investigator, with endorsement by the Steering Comm[illegible]mmittee (where applicable).

Any modifications to the study will be applied for all subsequent patients

11.2 Early Termination or Extension of the Study

The investigator (with Ethics Committee approval) may discontinue or extend the study at any time.

11.3 Confidentiality/Publication of Study Results

Interim and preliminary results should not be discussed or presented outside the Trial Group, unless authorised by the chair of the Trial Steering Committee. The investigators plan to publish the results in a peer-reviewed journal.

11.4 Retention of Records

All CRFs and all other documents associated with this study must be archived for at least 7 years following the completion of the trial, in accordance with TGA requirements.

11.5 Audits

For the purpose of compliance with ICH GCP notes for Guidance on Good Clinical Practice (CPMP/ICH/135/95), it may be necessary for a regulatory agency to conduct a site audit.

Random audits may be conducted throughout the trial at the discretion of the Trial Steering Committee.

12. ETHICAL PROCEDURES

12.1 Guidelines for Good Clinical Practice

This study is to be performed in accordance with ICH GCP notes for Guidance on Good Clinical Practice (CPMP/ICH/135/95).

12.2 Precautionary Advice

None specifically required.

12.3 Participant Information Sheet and Consent Form

The investigator or delegate will explain the study verbally to the patient. The patient will then be given a copy of the PI&CF and given an opportunity to read it and ask any questions of the investigator. The patient will be encouraged to obtain additional information about the study from an independent source. Once the patient is satisfied with the information they have received, has had an opportunity to ask questions and obtain additional information, and the

investigator is satisfied that the patient truly understands the nature of the study, the patient will be asked to sign the consent form.

The signing of the consent form must take place in front of a witness and that witness must also be satisfied that the patient has a good understanding of the study. Each patient's signed consent form will be retained by the investigator.

Patients will be advised that they are free to refuse to participate in, or to withdraw from the study at any time. The medical care provided will not be affected by agreement or refusal to participate in this study. The original Consent Form for each subject will be stored in the Investigators file and a copy of the consent form will be placed in the patient's medical record.

12.4 Ethics Committee

This protocol will be submitted to the Ethics Committee (or relevant regulatory body) at each site and their approval obtained.

13. AUTHORSHIP PLAN

RELIEF Trial
Authorship & Agreement

Target Journal: Lancet, New England Journal of Medicine, or JAMA

Planned Authorship: *The RELIEF Trial Investigators*

The trial will be described as a collaboration of the Australian and New Zealand College of Anaesthetists (ANZCA) Trials Group and the Australian and New Zealand Intensive Care Society (ANZICS) Clinical Trials Group.

The planned writing committee will include Paul Myles, Rinaldo Bellomo, Tomas Corcoran, Andrew Forbes, Philip Peyton, David Story, Chris Christophi, Andrew Davies, Kate Leslie, and Jonathan Serpell. This list may be extended or altered, according to a majority vote of the Trial Steering Committee.

Committee members and Site investigators at centres recruiting more than 250 patients will be offered co-authorship on at least one of the secondary publications. A more extensive participation and higher rate of patient enrolment may support a claim for authorship on the main publication (above), subject to a majority vote of the Trial Steering Committee.

Following acceptance for publication, all co-investigators (site investigators at each centre) can have access to all trial data if they would like to plan secondary analysis (and follow-up publication or presentation). A separate protocol should be developed and will require approval by the Trial Steering Committee before the presentation is made or submitted for publication.

An **Authorship Agreement** document will be produced before commencement of the trial, and all site investigators will be asked to sign their acknowledgement of this.

All site investigators listed in the appendix of the final publication(s) can be considered an author and so can list the publication(s) on their CVs.

Agreement to Participation

I have read the trial protocol and agree to conduct the study according to the procedures outlined, and in accordance with Good Clinical Research Practice (GCRP) guidelines. Any information related to this trial will be kept confidential until publication or presentation at a scientific meeting. I have read and accept the proposed authorship plan.

Site Coordinator (print): ……………………………….

Signature: ……………………………………   Date: ……/…../………

References

1. Myles P, Leslie K. Anaesthesia for major abdominal and urological surgery. Evidence-based Anaesthesia and Intensive Care [textbook] In: Møller A, Pederson T (eds). 2006:223-46.
2. SIGN. Postoperative management in adults. A practical guide to postoperative care for clinical staff. Scottish Intercollegiate Guidelines Network (SIGN). [Guideline]. 2004;77.
3. Rahbari NN, Zimmermann JB, Schmidt T, Koch M, Weigand MA, Weitz J. Meta-analysis of standard, restrictive and supplemental fluid administration in colorectal surgery. Br J Surg. 2009 Apr;96(4):331-41.
4. Bundgaard-Nielsen M, Secher NH, Kehlet H. 'Liberal' vs. 'restrictive' perioperative fluid therapy--a critical assessment of the evidence. Acta Anaesthesiol Scand. 2009 Aug;53(7):843-51.
5. Chong PC, Greco EF, Stothart D, Maziak DE, Sundaresan S, Shamji FM, et al. Substantial variation of both opinions and practice regarding perioperative fluid resuscitation. Can J Surg. 2009 Jun;52(3):207-14.
6. Hannemann P, Lassen K, Hausel J, Nimmo S, Ljungqvist O, Nygren J, et al. Patterns in current anaesthesiological peri-operative practice for colonic resections: a survey in five northern-European countries. Acta Anaesthesiol Scand. 2006 Oct;50(9):1152-60.
7. Weiser TG, Regenbogen SE, Thompson KD, Haynes AB, Lipsitz SR, Berry WR, et al. An estimation of the global volume of surgery: a modelling strategy based on available data. Lancet. 2008 Jul 12;372(9633):139-44.
8. Australian Institute of Health and Welfare A. Australian hospital statistics 2007-08. 2009.
9. Shires T, Williams J, Brown F. Acute change in extracellular fluids associated with major surgical procedures. Ann Surg. 1961 Nov;154:803-10.
10. Arkilic CF, Taguchi A, Sharma N, Ratnaraj J, Sessler DI, Read TE, et al. Supplemental perioperative fluid administration increases tissue oxygen pressure. Surgery. 2003 Jan;133(1):49-55.
11. Mythen MG, Webb AR. The role of gut mucosal hypoperfusion in the pathogenesis of post-operative organ dysfunction. Intensive Care Med. 1994;20(3):203-9.
12. Davies SJ, Wilson RJ. Preoperative optimization of the high-risk surgical patient. Br J Anaesth. 2004 Jul;93(1):121-8.
13. Holte K, Klarskov B, Christensen DS, Lund C, Nielsen KG, Bie P, et al. Liberal versus restrictive fluid administration to improve recovery after laparoscopic cholecystectomy: a randomized, double-blind study. Ann Surg. 2004 Nov;240(5):892-9.
14. Boyd O, Grounds RM, Bennett ED. A randomized clinical trial of the effect of deliberate perioperative increase of oxygen delivery on mortality in high-risk surgical patients. JAMA. 1993 Dec 8;270(22):2699-707.
15. Wilson J, Woods I, Fawcett J, Whall R, Dibb W, Morris C, et al. Reducing the risk of major elective surgery: randomised controlled trial of preoperative optimisation of oxygen delivery. BMJ. 1999 Apr 24;318(7191):1099-103.
16. Arieff AI. Fatal postoperative pulmonary edema: pathogenesis and literature review. Chest. 1999 May;115(5):1371-7.
17. Ruttmann TG, James MF, Aronson I. In vivo investigation into the effects of haemodilution with hydroxyethyl starch (200/0.5) and normal saline on coagulation. Br J Anaesth. 1998 May;80(5):612-6.
18. Ratner AJ, Lysenko ES, Paul MN, Weiser JN. Synergistic proinflammatory responses induced by polymicrobial colonization of epithelial surfaces. Proc Natl Acad Sci U S A. 2005 Mar 1;102(9):3429-34.
19. Lang K, Boldt J, Suttner S, Haisch G. Colloids versus crystalloids and tissue oxygen tension in patients undergoing major abdominal surgery. Anesth Analg. 2001 Aug;93(2):405-9 , 3rd contents page.
20. Lassen K, Soop M, Nygren J, Cox PB, Hendry PO, Spies C, et al. Consensus review of optimal perioperative care in colorectal surgery: Enhanced Recovery After Surgery (ERAS) Group recommendations. Arch Surg. 2009 Oct;144(10):961-9.

21. Lobo DN, Bostock KA, Neal KR, Perkins AC, Rowlands BJ, Allison SP. Effect of salt and water balance on recovery of gastrointestinal function after elective colonic resection: a randomised controlled trial. Lancet. 2002 May 25;359(9320):1812-8.

22. Tambyraja AL, Sengupta F, MacGregor AB, Bartolo DC, Fearon KC. Patterns and clinical outcomes associated with routine intravenous sodium and fluid administration after colorectal resection. World J Surg. 2004 Oct;28(10):1046-51; discussion 51-2.

23. Corcoran TR, JEJ. Clarke, S. Myles, PS. Oh, KM. Perioperative fluid management strategies in major surgery: a stratified meta-analysis. under peer review. 2011.

24. Rahbari NN, Zimmermann JB, Schmidt T, Koch M, Weigand MA, Weitz J. Meta-analysis of standard, restrictive and supplemental fluid administration in colorectal surgery. British Journal of Surgery. 2009;96(4):331-41.

25. Nisanevich V, Felsenstein I, Almogy G, Weissman C, Einav S, Matot I. Effect of intraoperative fluid management on outcome after intraabdominal surgery. Anesthesiology. 2005 Jul;103(1):25-32.

26. Kabon B, Akca O, Taguchi A, Nagele A, Jebadurai R, Arkilic CF, et al. Supplemental intravenous crystalloid administration does not reduce the risk of surgical wound infection. Anesth Analg. 2005 Nov;101(5):1546-53.

27. MacKay G, Fearon K, McConnachie A, Serpell MG, Molloy RG, O'Dwyer PJ. Randomized clinical trial of the effect of postoperative intravenous fluid restriction on recovery after elective colorectal surgery. Br J Surg. 2006 Dec;93(12):1469-74.

28. Vermeulen H, Hofland J, Legemate DA, Ubbink DT. Intravenous fluid restriction after major abdominal surgery: a randomized blinded clinical trial. Trials. 2009;10:50.

29. Finfer S, Bellomo R, Boyce N, French J, Myburgh J, Norton R. A comparison of albumin and saline for fluid resuscitation in the intensive care unit. N Engl J Med. 2004 May 27;350(22):2247-56.

30. Perner A, Haase N, Guttormsen AB, Tenhunen J, Klemenzson G, Aneman A, et al. Hydroxyethyl starch 130/0.42 versus Ringer's acetate in severe sepsis. N Engl J Med. 2012 Jul 12;367(2):124-34.

31. Myburgh JA, Finfer S, Bellomo R, Billot L, Cass A, Gattas D, et al. Hydroxyethyl Starch or Saline for Fluid Resuscitation in Intensive Care. N Engl J Med. 2012 Oct 17.

32. Marik PE, Baram M, Vahid B. Does central venous pressure predict fluid responsiveness? A systematic review of the literature and the tale of seven mares. Chest. 2008 Jul;134(1):172-8.

33. Peyton PJ, Chong SW. Minimally invasive measurement of cardiac output during surgery and critical care: a meta-analysis of accuracy and precision. Anesthesiology. 2010 Nov;113(5):1220-35.

34. Noblett SE, Snowden CP, Shenton BK, Horgan AF. Randomized clinical trial assessing the effect of Doppler-optimized fluid management on outcome after elective colorectal resection. Br J Surg. 2006 Sep;93(9):1069-76.

35. Conway DH, Mayall R, Abdul-Latif MS, Gilligan S, Tackaberry C. Randomised controlled trial investigating the influence of intravenous fluid titration using oesophageal Doppler monitoring during bowel surgery. Anaesthesia. 2002 Sep;57(9):845-9.

36. Wakeling HG, McFall MR, Jenkins CS, Woods WG, Miles WF, Barclay GR, et al. Intraoperative oesophageal Doppler guided fluid management shortens postoperative hospital stay after major bowel surgery. Br J Anaesth. 2005 Nov;95(5):634-42.

37. Futier E, Constantin JM, Petit A, Chanques G, Kwiatkowski F, Flamein R, et al. Conservative vs restrictive individualized goal-directed fluid replacement strategy in major abdominal surgery: A prospective randomized trial. Arch Surg. 2010 Dec;145(12):1193-200.

38. Walsh SR, Tang T, Bass S, Gaunt ME. Doppler-guided intra-operative fluid management during major abdominal surgery: systematic review and meta-analysis. Int J Clin Pract. 2008 Mar;62(3):466-70.

39. Mowatt G, Houston G, Hernandez R, de Verteuil R, Fraser C, Cuthbertson B, et al. Systematic review of the clinical effectiveness and cost-effectiveness of oesophageal Doppler

monitoring in critically ill and high-risk surgical patients. Health Technol Assess. 2009 Jan;13(7):iii-iv, ix-xii, 1-95.
40. Davies SJ, Yates D, Wilson RJT. Dopexamine Has No Additional Benefit in High-Risk Patients Receiving Goal-Directed Fluid Therapy Undergoing Major Abdominal Surgery. Anesthesia & Analgesia. 2011;112(1):130-8.
41. Hamilton MA, Cecconi M, Rhodes A. A systematic review and meta-analysis on the use of preemptive hemodynamic intervention to improve postoperative outcomes in moderate and high-risk surgical patients. Anesth Analg. 2011 Jun;112(6):1392-402.
42. Challand C, Struthers R, Sneyd JR, Erasmus PD, Mellor N, Hosie KB, et al. Randomized controlled trial of intraoperative goal-directed fluid therapy in aerobically fit and unfit patients having major colorectal surgery. Br J Anaesth. 2012 Jan;108(1):53-62.
43. Bellamy MC. Wet, dry or something else? Br J Anaesth. 2006 Dec;97(6):755-7.
44. Varadhan KK, Neal KR, Dejong CH, Fearon KC, Ljungqvist O, Lobo DN. The enhanced recovery after surgery (ERAS) pathway for patients undergoing major elective open colorectal surgery: a meta-analysis of randomized controlled trials. Clin Nutr. 2010 Aug;29(4):434-40.
45. Myles PS, Devereaux PJ. Pros and cons of composite endpoints in anesthesia trials. Anesthesiology. 2010 Oct;113(4):776-8.
46. Myles PS, Weitkamp B, Jones K, Melick J, Hensen S. Validity and reliability of a postoperative quality of recovery score: the QoR-40. Br J Anaesth. 2000 Jan;84(1):11-5.
47. Myles PS, Hunt JO, Fletcher H, Solly R, Woodward D, Kelly S. Relation between quality of recovery in hospital and quality of life at 3 months after cardiac surgery. Anesthesiology. 2001 Oct;95(4):862-7.
48. Myles P, Smith J, Knight J, Cooper D, Silbert B, McNeil J, et al. Aspirin and Tranexamic Acid for Coronary Artery Surgery (ATACAS) Trial: Rationale and design. American Heart Journal. 2008;155(2):224-30.
49. Myles PS, Leslie K, Peyton P, Paech M, Forbes A, Chan MT, et al. Nitrous oxide and perioperative cardiac morbidity (ENIGMA-II) Trial: rationale and design. Am Heart J. 2009 Mar;157(3):488-94 e1.
50. Myles PS, Bellomo R. A pivotal trial of fluid therapy for major abdominal surgery: need and equipoise. Crit Care Resusc. 2011 Dec;13(4):278-80.
51. Prowle JR, Chua HR, Bagshaw SM, Bellomo R. Clinical review: Volume of fluid resuscitation and the incidence of acute kidney injury - a systematic review. Crit Care. 2012 Aug 7;16(4):230.
52. Hubner M, Schafer M, Demartines N, Muller S, Maurer K, Baulig W, et al. Impact of Restrictive Intravenous Fluid Replacement and Combined Epidural Analgesia on Perioperative Volume Balance and Renal Function Within a Fast Track Program. J Surg Res. 2010 Sep 27.
53. Rahbari NN, Weitz J, Hohenberger W, Heald RJ, Moran B, Ulrich A, et al. Definition and grading of anastomotic leakage following anterior resection of the rectum: a proposal by the International Study Group of Rectal Cancer. Surgery. 2010 Mar;147(3):339-51.
54. Horan T, Andrus M, Dudeck M. CDC/NHSN surveillance definition of health care–associated infection and criteria for specific types of infections in the acute care setting. American Journal of Infection Control. 2008;36(5):309-32.
55. Bellomo R, Ronco C, Kellum JA, Mehta RL, Palevsky P. Acute renal failure - definition, outcome measures, animal models, fluid therapy and information technology needs: the Second International Consensus Conference of the Acute Dialysis Quality Initiative (ADQI) Group. Crit Care. 2004 Aug;8(4):R204-12.
56. Rigg JR, Jamrozik K, Myles PS, Silbert BS, Peyton PJ, Parsons RW, et al. Epidural anaesthesia and analgesia and outcome of major surgery: a randomised trial. Lancet. 2002 Apr 13;359(9314):1276-82.
57. Hebert PC, Wells G, Blajchman MA, Marshall J, Martin C, Pagliarello G, et al. A multicenter, randomized, controlled clinical trial of transfusion requirements in critical care.

Transfusion Requirements in Critical Care Investigators, Canadian Critical Care Trials Group. N Engl J Med. 1999 Feb 11;340(6):409-17.
58. Hebert PC, Yetisir E, Martin C, Blajchman MA, Wells G, Marshall J, et al. Is a low transfusion threshold safe in critically ill patients with cardiovascular diseases? Crit Care Med. 2001 Feb;29(2):227-34.
59. Scheingraber S, Rehm M, Sehmisch C, Finsterer U. Rapid saline infusion produces hyperchloremic acidosis in patients undergoing gynecologic surgery. Anesthesiology. 1999 May;90(5):1265-70.
60. Shaw AD, Bagshaw SM, Goldstein SL, Scherer LA, Duan M, Schermer CR, et al. Major complications, mortality, and resource utilization after open abdominal surgery: 0.9% saline compared to Plasma-Lyte. Ann Surg. 2012 May;255(5):821-9.
61. Yunos NM, Bellomo R, Hegarty C, Story D, Ho L, Bailey M. Association between a chloride-liberal vs chloride-restrictive intravenous fluid administration strategy and kidney injury in critically ill adults. JAMA. 2012 Oct 17;308(15):1566-72.
62. Prowle JR, Liu YL, Licari E, Bagshaw SM, Egi M, Haase M, et al. Oliguria as predictive biomarker of acute kidney injury in critically ill patients. Crit Care. 2011;15(4):R172.
63. Zacharias M, Conlon NP, Herbison GP, Sivalingam P, Walker RJ, Hovhannisyan K. Interventions for protecting renal function in the perioperative period. Cochrane Database Syst Rev. 2008(4):CD003590.
64. Mamidanna R, Burns EM, Bottle A, Aylin P, Stonell C, Hanna GB, et al. Reduced Risk of Medical Morbidity and Mortality in Patients Selected for Laparoscopic Colorectal Resection in England: A Population-Based Study. Arch Surg. 2011 Nov 21.
65. Monk T, Saini V, Weldon B, Sigl J. Anesthetic management and one-year mortality after noncardiac surgery. Anesth Analg. 2005;100:4-10.
66. Myles PS, Peyton P, Silbert B, Hunt J, Rigg JR, Sessler DI. Perioperative epidural analgesia for major abdominal surgery for cancer and recurrence-free survival: randomised trial. BMJ. 2011;342:d1491.
67. Graham AM, Myles PS, Leslie K, Chan MT, Paech MJ, Peyton P, et al. A cost-benefit analysis of the ENIGMA trial. Anesthesiology. 2011 Aug;115(2):265-72.

RELIEF Statistical Analysis Plan

August 29, 2017

We will apply the intention to treat principle, analysing all participants who are enrolled, randomised and undergo induction of general anaesthesia for eligible surgery. Patients are followed for the duration of the trial, unless they withdraw consent, for which we will use their data up until the time of withdrawal of consent.

ENDPOINT DEFINITIONS

Primary endpoint

The primary end point of the trial is disability-free survival at 1 year after surgery. Disability is defined as a persistent (at least 6 months) impairment in health status, as measured by the 12-item WHODAS score, of at least 24 points when using response scores of 1–5 for each item, reflecting a disability level of at least 25% and being the threshold point between 'disabled' and 'not disabled' as per WHO guidelines. If a single item is missing at an assessment, the mean value of the remaining items will be assigned to the missing item. If more than one item is missing the score will not be calculated for that assessment.

With WHODAS assessments being made at (baseline and) 30 days, 3 months, 6 months and 12 months, post-operative disability that persists for at least 6 months is able to be observed to be commencing at the 30 day assessment, the 3 month assessment, or the 6 month assessment. For example, persistent disability commencing at 3 months requires the initial observation of disability (WHODAS >=24) at 3 months which is sustained at each of the 6 and 12 month assessments. Missing WHODAS assessments in patients known to be alive will not be imputed in the primary analysis. Persistent disability observed to commence at the 30 day assessment will be assumed to be related to surgery and will be assigned an onset date of 0.10 days post-surgery. Onset of disability at 3 or 6 months postoperatively will typically be after an incident/illness in the postoperative follow-up period, and for these events the self-reported date of such onset will be utilised. If no such event is documented, then the current time point (interview date) will be used.

The time to the primary endpoint is defined as the time of the onset of persistent (>= 6-month) disability or death, whichever occurs first. Time at risk will commence at start of surgery to accommodate the potential for intraoperative mortality. Patients not experiencing the primary endpoint event will be censored at their date of last contact.

Two supplementary approaches will be utilised to assess sensitivity to handling of missing WHODAS assessments for subjects known to be alive at those assessment times: (a) they will be given a disabled score (WHODAS of 24), and (b) they will be imputed using information from baseline and post-baseline variables (see statistical analysis methods).

Alternative 'new onset disability' definition of the primary endpoint

An additional sensitivity analysis will done for an alternative definition of persistent disability, considered as 'new onset' persistent disability, defined as an increase from baseline of >=4 points in WHODAS scores that persists for at least 6 months. The definition of time to the first of new-onset persistent disability or death will use the same principles as for the primary endpoint.

Secondary endpoints

1. Death/survival: all-cause mortality at 90 days, and survival up to 12 months after surgery.

2. A composite (pooled) and individual incidence of 30-day mortality and major septic complications, where the latter is defined as the composite of sepsis, surgical site infection, anastomotic leak and pneumonia at 30 days post-surgery. [Detailed clinical definitions are provided in the Protocol]

3. Acute Kidney Injury (AKI): according to the Kidney Disease: Improving Global Outcomes group criteria, but not urine output—for Stage 2 or worse AKI defined as at least twofold increase in creatinine, or estimated glomerular filtration rate decrease >50%.

   Since a restrictive intravenous fluid regimen may artificially elevate serum creatinine due to a smaller dilutional effect from less intravenous fluids, we will calculate adjusted creatinine following the approach of Liu (2011, Reference 1 below), where *adjusted creatinine = serum creatinine × (1 + cumulative fluid balance/total body water)*, and assuming that total body water is 60% of body weight, expressed in mL. Serum creatinine is measured on days 1 and 3 and the maximum value in the patient's hospital stay. We will apply adjustments to creatinine levels at days 1 and 3 only. Fluid intake will be accumulated using IV fluids administered intraoperatively, in recovery, and on days 1 to 3 postoperatively, plus the volume of any blood transfusions administered. Fluid outputs from the time of surgery to Day 1 post-surgery will be accumulated using the recorded urine outputs, blood losses and volumes in surgical drains. Missing fluid output components will be imputed to prevent adjustment factors being missing when creatinine levels are present. Fluid outputs on days 2 and 3 are not recorded, so these will be estimated under the assumption of a net fluid balance of zero on each of days 2 and 3; this will form the principal analysis. Sensitivity to this assumption will be assessed using two alternatives: (a) assuming a zero cumulative fluid balance at day 3, and (b) assuming the ratio of intake to outputs up to day 1 persists on days 2 and 3. These two assumptions enclose that of the principal analysis. We will also report the use of renal replacement therapy up to 90 days after surgery; and delta-creatinine, defined as the difference between the maximum (fluid-adjusted) postoperative serum creatinine level and the preoperative serum creatinine level.

4. Pulmonary oedema: documented evidence of respiratory distress or impaired oxygenation and radiological evidence of pulmonary oedema.

5. Duration of mechanical ventilation: Defined as additive over all episodes up to 90 days after surgery. This will be reported as (a) the proportion of patients requiring ventilation; and (b) the duration of ventilation in patients receiving ventilation.

6. Inflammation: plasma C reactive protein concentration on day 3 after surgery.

7. Tissue perfusion marker: peak serum lactate concentration within 24 hours of surgery.

8. Any blood transfusion: including red cell, fresh frozen plasma or platelet transfusion, from the initiation of surgery; and quantity of transfusion in patients receiving each product.

9. Unplanned admission to HDU/ICU within 30 days of surgery.

10. Total HDU/ICU stay in patients admitted to HDU/ICU, including initial admission and readmission duration up to day 30

11. Total hospital stay, including any readmission up to day 30.

12. Quality of recovery: QoR-15 score on days 1, 3 and 30.

13. The rates of serious adverse events, and severity of adverse events (mild, moderate, severe), classified by organ system.

STATISTICAL ANALYSES

Primary endpoint: disability-free survival

Disability free survival will be displayed with Kaplan-Meier plots, and described with event-free proportions in each treatment arm obtained from these plots at days 1, 30, 90, 180 and 365 days post-surgery. Comparison of overall time to events between treatment arms will be made using the log rank test and the Cox proportional hazards model to provide a hazard ratio and 95% CI. Assessment of proportionality of hazards will be based on tests using Schoenfeld residuals. The principal analysis will not impute missing WHODAS measurements for patients known to be alive at those assessment times. The first sensitivity analysis will impute all missing WHODAS assessments for subjects known to be alive at those assessment times by giving them a disabled score (WHODAS of 24). A second sensitivity analysis will impute the missing WHODAS assessments using multiple imputation, with the imputation model employing baseline and post-baseline information predictive of missingness or WHODAS scores, separately in each treatment arm, with results combined across imputations using Rubin’s rules.

Alternative 'new onset' definition of the primary endpoint

Analysis of disability-free survival based on the `new onset' persistent disability definition will follow the same approach as for the primary endpoint.

Time to death

Analysis of time to death will follow the same approach as for the primary endpoint.

Other outcomes

Secondary outcomes measured on a binary scale (1, 2, 3, 4, 8, 9) will be summarised using proportions in each treatment arm and analysed using binomial regression with a logarithmic link to estimate Risk Ratios with 95% CIs and p-values, or exact logistic regression to approximate Risk ratios if the number of events in either arm is fewer than 10. Should there be convergence difficulties with log-binomial regression, a log-Poisson model will be employed with robust standard errors.

Duration and length of stay outcomes (5, 10, 11) will be summarised using medians and interquartile ranges, and compared across treatment arms using the Wilcoxon– Breslow–Gehan test, with length of stay in hospital and in intensive care censored at 30 days, and with in-hospital deaths assigned the highest length of stay.

Outcomes measured on a continuous or semi-continuous scale (6, 7, 8, 12) will be summarised by means and standard deviations if reasonably symmetrically distributed and compared between treatment arms using linear regression with robust standard errors. Skewed outcomes will be summarised by medians and interquartile ranges; right skewed outcomes will be log-transformed prior to analysis using linear regression, and left skewed outcomes will be analysed using median regression with robust standard errors.

Additional sensitivity analyses

Sensitivity analyses for all outcomes will use regression models with additional adjustment for the stratification variables of site and planned HDU/ICU destination status, plus any variables exhibiting substantial imbalance across treatment arms at baseline.

Sensitivity to missing outcome data will be performed using multiple imputation if the proportion of missing data for the particular outcome is >5%. These analyses will use multiple imputation, employing imputation models with baseline and auxiliary post-baseline variables, and results combined across imputations using Rubin's rule.

Subgroup analyses

Planned subgroup analyses will assess heterogeneity of treatment effects of the primary endpoint across patient sex, age groups (approximate quartiles), country, bowel surgery (yes/no) and intraoperative use of any goal-directed techniques (yes/no). The latter include invasive or non-invasive cardiac output, stroke volume or pulse pressure variation and oesophageal Doppler, but exclude central venous pressure monitoring.

Additional prespecified subgroups will be tested for heterogeneity of effect, and their results considered exploratory: BMI categories (defined as underweight <18.5, normal 18.5-25, overweight 25-30, obese 30-35, super obese >35), ASA physical status (1/2, 3, 4), pre-operative planned HDU/ICU destination status, duration of surgery (approximate quartiles), and pre-operative planned use of a goal directed device (excluding CVP monitoring).

Additional analyses of the above subgroups will be performed for the endpoints of new-onset disability, composite of 30 day mortality and septic complications, and acute kidney injury.

For these analyses, we will undertake tests for interaction by adding treatment-by-covariate terms to the regression models specified for the main analyses of each outcome.

SAMPLE SIZE RE-ESTIMATION

The original sample size calculation was as follows: Assuming a 12 month disability-free survival probability of 65%, 2650 patients were required to detect a hazard ratio of 0.80 with 90% power using the Freedman method for the sample size for a log rank test. Correspondingly, 850 events were expected to be observed. The sample size was inflated to a total of 2800 patients to account for withdrawals and loss to follow-up.

A sample size reassessment of the assumed primary endpoint event rate was performed after 2578 patients had been randomised. At that time there were 300 primary endpoint events with a 12 month event rate of approximately 15%. Increasing the target sample size to 3000 patients under this same event rate was expected to yield approximately 380 events and afford 80% power to detect a hazard ratio of 0.75.

REFERENCES

1. Liu KD, Thompson BT, Ancukiewicz M, et al. Acute kidney injury in patients with acute lung injury: impact of fluid accumulation on classification of acute kidney injury and associated outcomes. Crit Care Med 2011;39:2665-71
