# Supplementary material for: Association of Obesity With Septic Complications After Major Abdominal Surgery: A Secondary Analysis of the RELIEF Randomized Clinical Trial
Source: JAMA Netw Open. 2019 Nov 27;2(11):e1916345. doi: 10.1001/jamanetworkopen.2019.16345 (PMC6902846; doi:10.1001/jamanetworkopen.2019.16345)
Supplement: Supplement 2. — eTable 1. Participants’ Eligibility Criteria eTable 2. Results From Regression Analyses for All Outcomes With Adjusted Adiposity Variables eFigure 1. Histograms of Waist Circumference, Waist-to-Hip Ratio, and BMI for 2755 Participants eFigure 2. Fitted Risk Prediction Model for Disability or Death to 90 Days eFigure 3. Fitted Risk Prediction Model for Septic Complications or Death to 30 Days eFigure 4. Fitted Risk Prediction Model for Any Major Septic Complication eFigure 5. Fitted Risk Prediction Model for Pulmonary Edema eFigure 6. Fitted Risk Prediction Model for Acute Kidney Injury eFigure 7. Fitted Risk Prediction Model for Unplanned ICU Admission to 30 Days [file jamanetwopen-2-e1916345-s002.pdf]

Supplementary Online Content

Gurunathan U, Rapchuk IL, Dickfos M, et al. Association of obesity with septic complications after major abdominal surgery: a secondary analysis of the RELIEF randomized clinical trial. *JAMA Netw Open*. 2019;2(11):e1916345. doi:10.1001/jamanetworkopen.2019.16345

eTable 1. Participants' Eligibility Criteria
eTable 2. Results From Regression Analyses for All Outcomes With Adjusted Adiposity Variables
eFigure 1. Histograms of Waist Circumference, Waist-to-Hip Ratio, and BMI for 2755 Participants
eFigure 2. Fitted Risk Prediction Model for Disability or Death to 90 Days
eFigure 3. Fitted Risk Prediction Model for Septic Complications or Death to 30 Days
eFigure 4. Fitted Risk Prediction Model for Any Major Septic Complication
eFigure 5. Fitted Risk Prediction Model for Pulmonary Edema
eFigure 6. Fitted Risk Prediction Model for Acute Kidney Injury
eFigure 7. Fitted Risk Prediction Model for Unplanned ICU Admission to 30 Days

This supplementary material has been provided by the authors to give readers additional information about their work.

© 2019 Gurunathan U et al. *JAMA Network Open.*

eTable 1: Participants' Eligibility Criteria

Inclusion criteria:

Adults 18 years and older undergoing elective major abdominal surgery of

a. atleast 2 hours duration
b. hospital stay of atleast 3 days and
c. at increased risk of postoperative complications, defined as atleast one of the following criteria:
    i. age ≥70 years,
    ii. known or documented history of coronary artery disease,
    iii. known or documented history of heart failure,
    iv. diabetes currently treated with an oral hypoglycemic agent and/or insulin,
    v. preoperative serum creatinine >200 μmol/L (>2.8 mg/dl),
    vi. morbid obesity (BMI ≥35 kg/m²),
    vii. preoperative serum albumin <30 g/L,
    viii. anerobic threshold (if done) <12 mL/kg/min,
    ix. r) two or more of the following risk factors: American Society of Anesthesiologists (ASA) physical status 3 or 4, chronic respiratory disease, obesity (BMI 30-35 kg/m²), aortic or peripheral vascular disease, preoperative hemoglobin <100 g/L, preoperative serum creatinine 150-199 μ mol/L (>1.7 mg/dl), anerobic threshold (if done) 12-14 mL/kg/min.

Exclusion criteria:

a. urgent or time-critical surgery,
b. ASA physical status 5
c. pulmonary or cardiac surgery
d. liver resection
e. minor or intermediate surgery
f. end-stage kidney failure requiring dialysis

© 2019 Gurunathan U et al. *JAMA Network Open.*

eTable 2. Results From Regression Analyses for All Outcomes With Adjusted Adiposity Variables

**Outcomes**	**Adiposity variable**	**N**	**Adjusted OR for each SD increase in the adiposity variable***	**95% CI**	**P-value**
Composite 30-day mortality or septic complication	Waist circumference	2636	**1.58**[a**, b**]	1.25 - 2.01	<0.001
	WHR		0.96	0.84 - 1.10	0.552
	BMI		0.90	0.72 - 1.12	0.324
Composite disability event or death	Waist circumference	2661	1.37	1.04 - 1.81	0.024
	WHR		1.01	0.87 - 1.16	0.936
	BMI		0.91	0.70 - 1.18	0.457
Septic complications	Waist circumference	2636	**1.57** [a**, b*]	1.24 - 2.00	<0.001
	WHR		0.97	0.85 - 1.11	0.679
	BMI		0.90	0.72 - 1.13	0.371
Pulmonary edema	Waist circumference	1695	1.53	0.70 - 3.34	0.282
	WHR		1.11	0.77 - 1.60	0.566
	BMI		1.11	0.51 - 2.44	0.786
Acute kidney injury	Waist circumference	2598	1.52	1.00 - 2.30	0.048
	WHR		1.15	0.93 - 1.41	0.203
	BMI		0.79	0.52 - 1.21	0.282
Unplanned ICU	Waist circumference	2611	1.25	0.91 - 1.71	0.177
	WHR		1.08	0.91 - 1.28	0.372
	BMI		1.58	0.58 - 1.09	0.152

WHR: waist-to-hip ratio, BMI: body mass index, ICU: intensive care unit
**From multivariable models including all three adiposity variables and adjusting for age, sex, smoking status, study center and treatment arm.*

*Superscripts: a = comparison of Waist circumference with WHR, b= comparison of Waist circumference with BMI; ** is P<0.01, * is 0.01 <= P < 0.05*
*Example: Composite outcome: a**, b* means Waist vs WHR P<0.01, Waist vs BMI 0.01<=P<0.05*

© 2019 Gurunathan U et al. *JAMA Network Open.*

e Figure 1. Histograms of Waist Circumference, Waist-to-Hip Ratio, and BMI for 2755 Participants

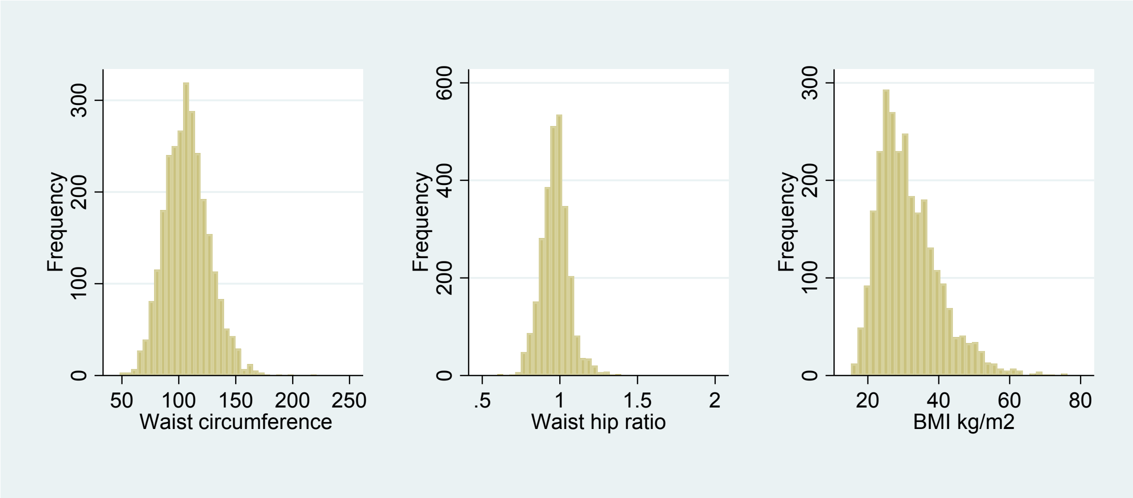

BMI: Body Mass Index

© 2019 Gurunathan U et al. *JAMA Network Open.*

eFigure 2. Fitted Risk Prediction Model for Disability or Death to 90 Days

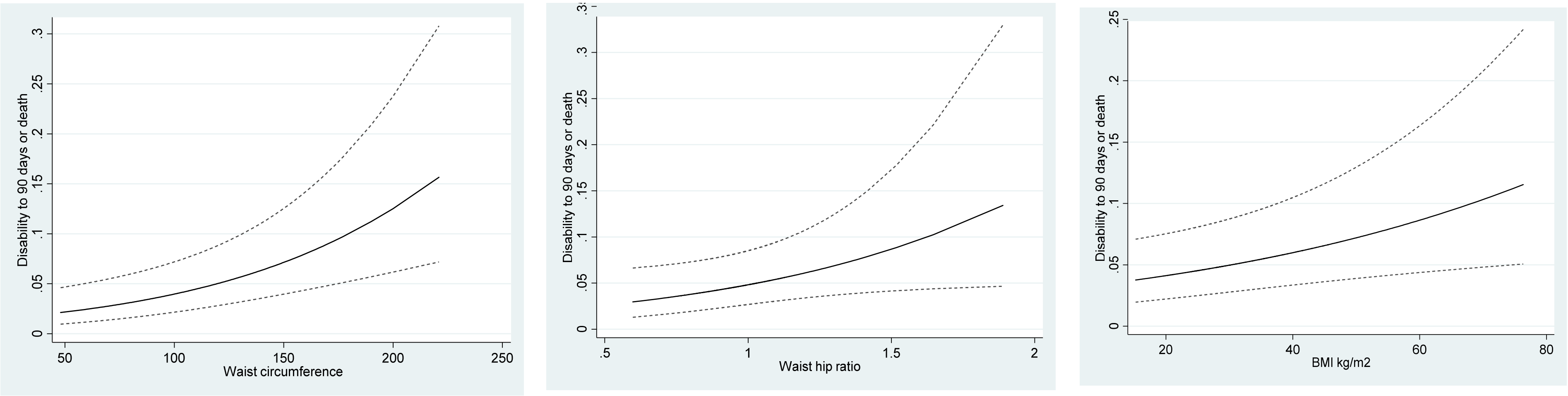

[#] *Graphs indicate the model for study center no:1*

© 2019 Gurunathan U et al. *JAMA Network Open.*

eFigure 3. Fitted Risk Prediction Model for Septic Complications or Death to 30 Days

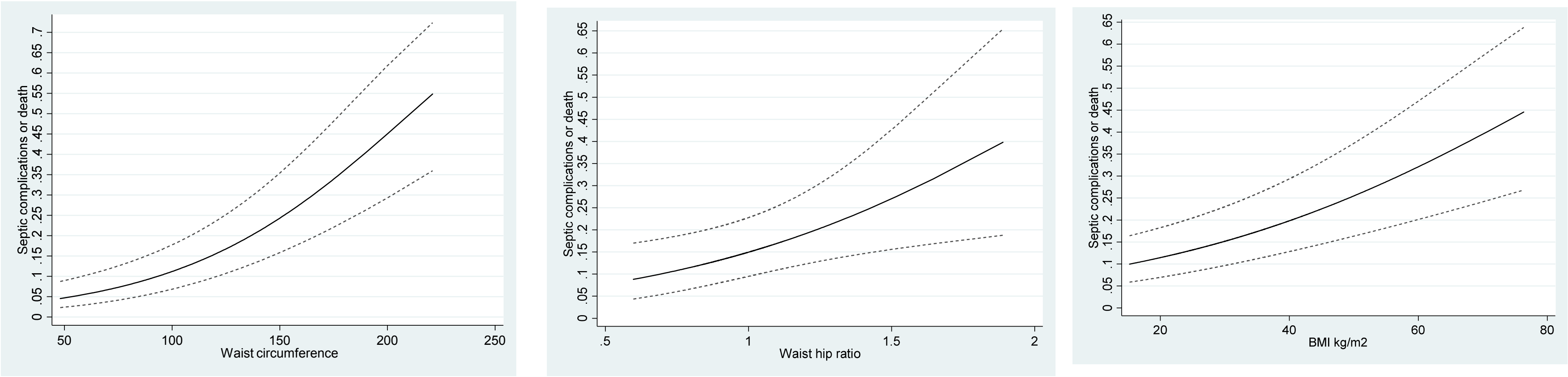

© 2019 Gurunathan U et al. *JAMA Network Open.*

eFigure 4. Fitted Risk Prediction Model for Any Major Septic Complication

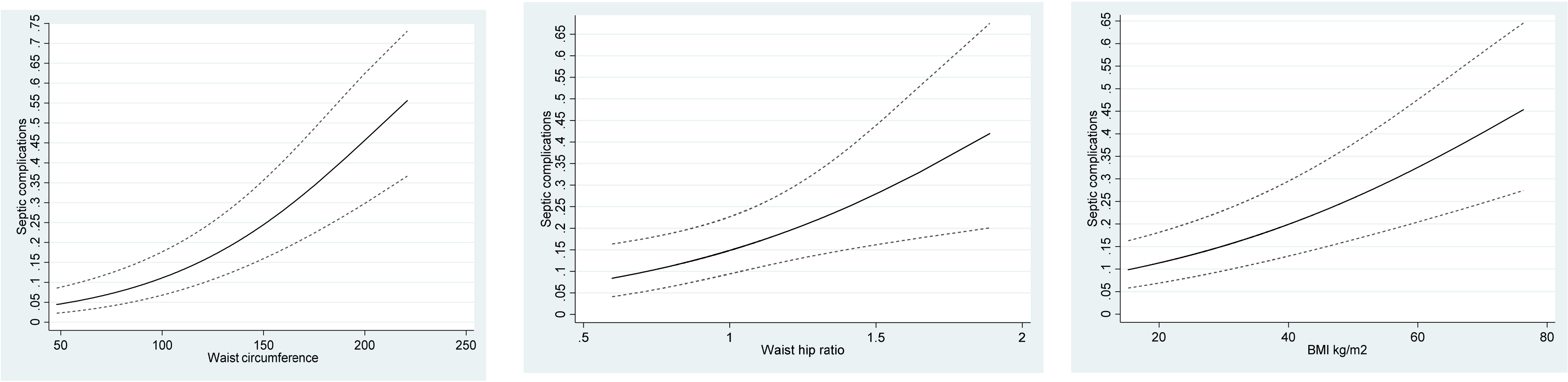

© 2019 Gurunathan U et al. *JAMA Network Open.*

eFigure 5. Fitted Risk Prediction Model for Pulmonary Edema

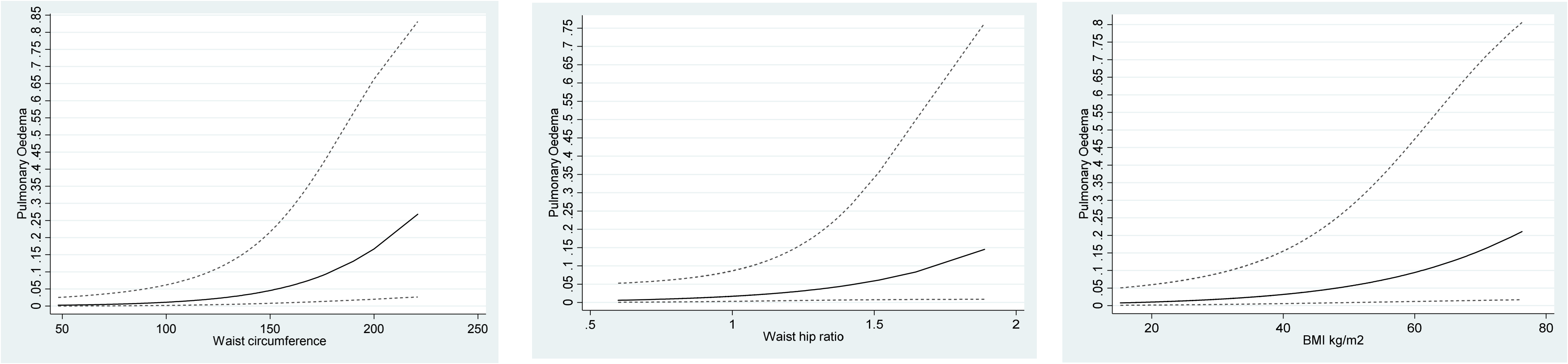

© 2019 Gurunathan U et al. *JAMA Network Open.*

eFigure 6. Fitted Risk Prediction Model for Acute Kidney Injury

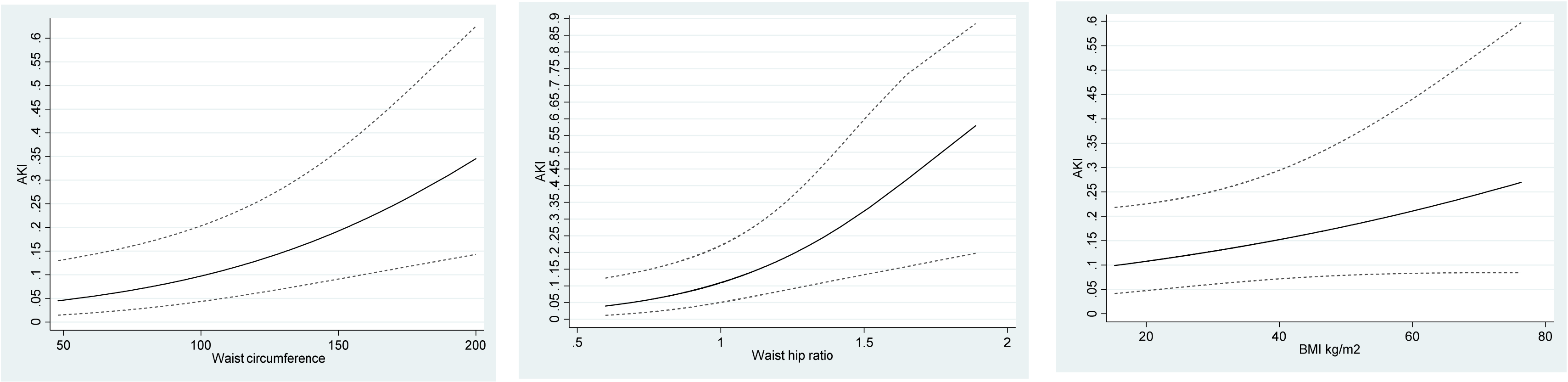

© 2019 Gurunathan U et al. *JAMA Network Open.*

eFigure 7. Fitted Risk Prediction Model for Unplanned ICU Admission to 30 Days

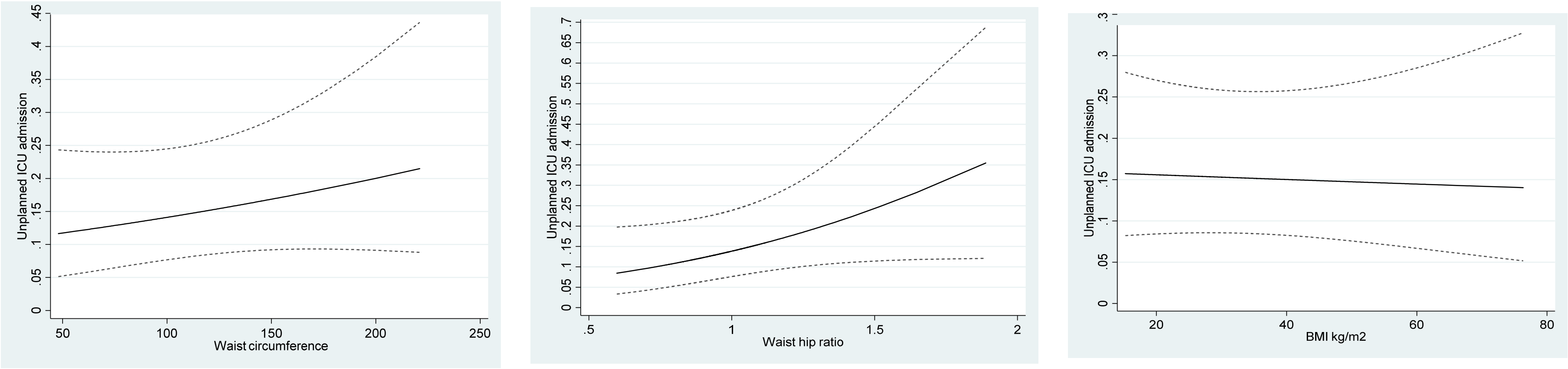

© 2019 Gurunathan U et al. *JAMA Network Open.*
